# Regeneration of glomerular metabolism and function by podocyte pyruvate kinase M2 in diabetic nephropathy

**DOI:** 10.1172/jci.insight.155260

**Published:** 2022-03-08

**Authors:** Jialin Fu, Takanori Shinjo, Qian Li, Ronald St-Louis, Kyoungmin Park, Marc G. Yu, Hisashi Yokomizo, Fabricio Simao, Qian Huang, I-Hsien Wu, George L. King

**Affiliations:** 1 Dianne Nunnally Hoppes Laboratory for Diabetes Complications, Joslin Diabetes Center and; 2 Joslin Diabetes Center, Harvard Medical School, Boston, Massachusetts, USA.

**Keywords:** Endocrinology, Metabolism, Chronic kidney disease, Diabetes, Glucose metabolism

## Abstract

Diabetic nephropathy (DN) arises from systemic and local changes in glucose metabolism and hemodynamics. We have reported that many glycolytic and mitochondrial enzymes, such as pyruvate kinase M2 (PKM2), were elevated in renal glomeruli of DN-protected patients with type 1 and type 2 diabetes. Here, mice with PKM2 overexpression specifically in podocytes (PPKM2Tg) were generated to uncover the renal protective function of PPKM2Tg as a potential therapeutic target that prevented elevated albumin/creatinine ratio (ACR), mesangial expansion, basement membrane thickness, and podocyte foot process effacement after 7 months of streptozotocin-induced (STZ-induced) diabetes. Furthermore, diabetes-induced impairments of glycolytic rate and mitochondrial function were normalized in diabetic PPKM2Tg glomeruli, in concordance with elevated *Ppargc1a* and *Vegf* expressions. Restored VEGF expression improved glomerular maximal mitochondrial function in diabetic PPKM2Tg and WT mice. Elevated VEGF levels were observed in the glomeruli of DN-protected patients with chronic type 1 diabetes and clinically correlated with estimated glomerular filtration (GFR) — but not glycemic control. Mechanistically, the preservations of mitochondrial function and VEGF expression were dependent on tetrameric structure and enzymatic activities of PKM2 in podocytes. These findings demonstrate that PKM2 structure and enzymatic activation in podocytes can preserve the entire glomerular mitochondrial function against toxicity of hyperglycemia via paracrine factors such as VEGF and prevent DN progression.

## Graphical abstract

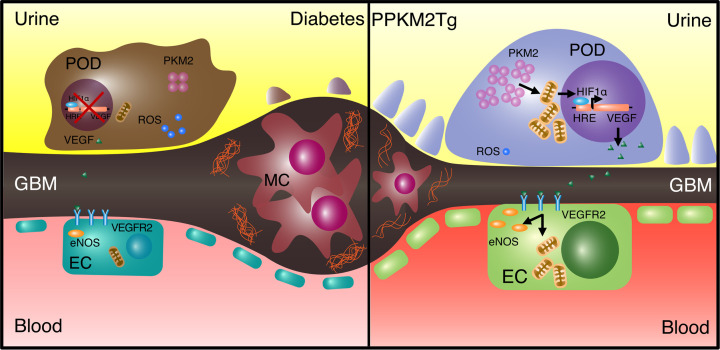

## Introduction

Diabetic nephropathy (DN) is the major cause of end-stage kidney disease (ESKD) in people with diabetes, affecting 30% of patients with diabetes (1–4). Multiple risk factors are associated with DN, including hypertension, hyperglycemia, insulin resistance, dyslipidemia, and familial clustering (5–10). In people with type 1 diabetes (T1D), hyperglycemia is the major risk factor for DN since glycemic control can prevent and delay its progression (10–13).

Thus, there is interest in identifying mechanisms of toxic effects by hyperglycemia, such as the disruption of filtration barrier due to abnormalities of glomerular podocytes, endothelial and mesangial cells, and glomerular basement membranes (GBM) (5). Among glomerular cells, podocyte dysfunction and apoptosis have generated a great deal of interest, since their pedicels are critical for the formation and maintenance of glomerular filtration barrier (14). This study is focused on the regulation of glycolysis and mitochondrial metabolism of podocytes and its effects on whole glomerular metabolism and pathology in normal and hyperglycemic conditions.

Podocyte loss in DN is related to the duration and severity of hyperglycemia, and it correlates with the progression of DN. A potential explanation for the close association of podocyte loss and declining glomerular filtration (GFR) is podocytes’ contributions to the metabolism and survival of glomerular cells by cytokines and growth factors expression and secretion. Eremina et al. reported that deletion of VEGF in podocytes resulted in collapsing of glomeruli and renal failure in mice (15). However, in diabetes, the changes in glomerular VEGF expressions are bimolded with elevation in initial stages of diabetes and significant reductions after chronic duration (16–18).

To clarify the mechanism of persistent hyperglycemia, we have been investigating the Joslin 50 Year Medalist Study, which is a cohort of people with long duration (≥50 years) of T1D and mostly protected from DN (19). In these individuals (*n =* 1023), only 13% have significant DN, which did not correlate with glycemic control (19), as opposed to other cohorts with T1D or T2D (13, 20, 21). Proteomic analysis of glomeruli from Medalists, or those from the Joslin 50 Year Medalist Study, demonstrated elevated glycolytic and mitochondrial enzymes such as pyruvate kinase M2 (PKM2) in DN-protective patients (22). These findings were confirmed in people with diabetes of shorter durations (23). To uncover the potential protective function of PKM2 specifically on podocytes against hyperglycemia, we generated mice with PKM2 overexpression specifically in podocytes (PPKM2Tg) and characterized its regulation of glomerular metabolism, mitochondrial functions, and VEGF expression in the development of DN, with correlation of glomerular VEGF expression to renal functions in Medalists with T1D.

## Results

### Characterization of PPKM2Tg mice.

**Figure 1:**
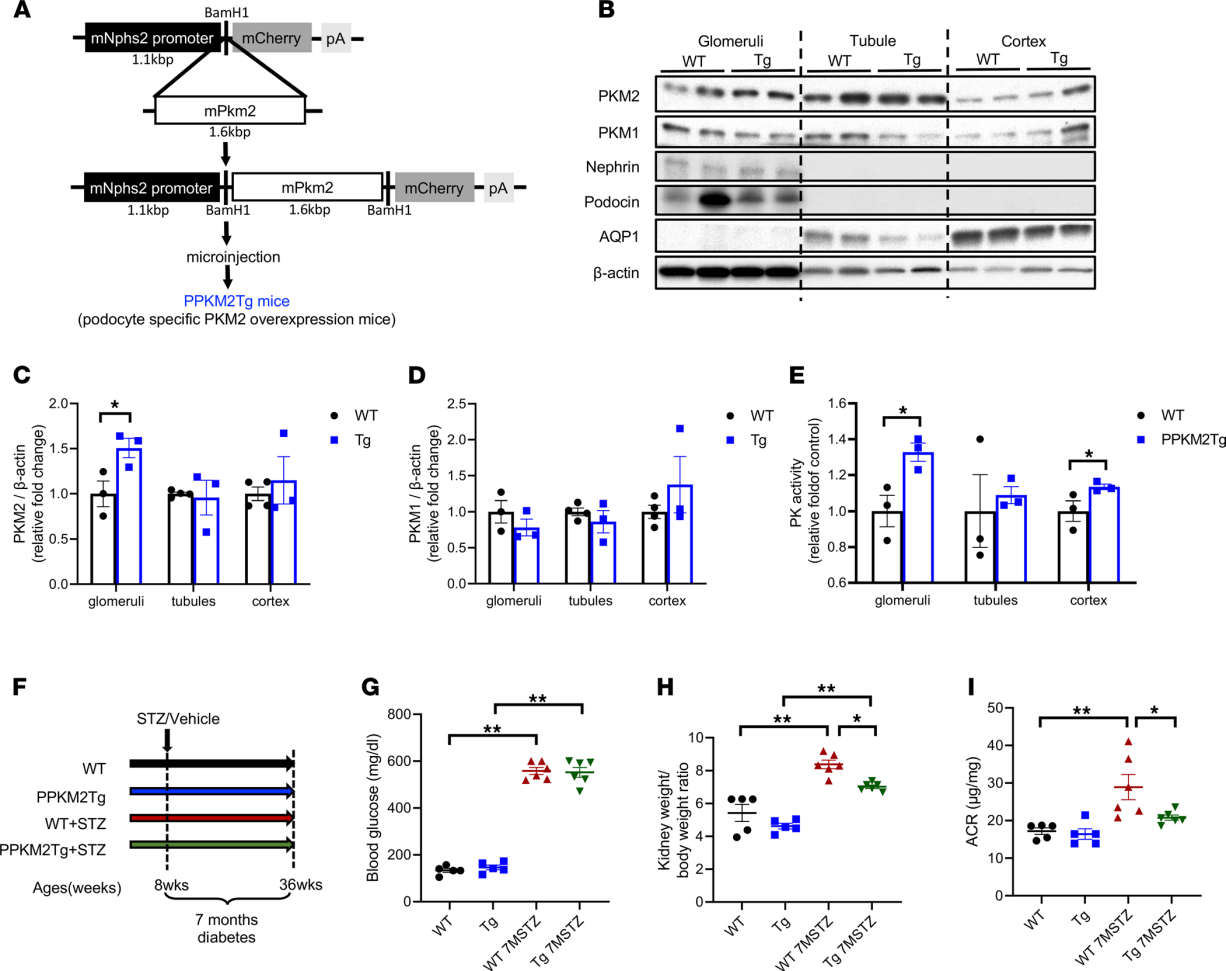
PPKM2Tg mice were resistant to diabetes-induced kidney injury. (**A**) Schema of PPKM2Tg mice generation. The complete mouse cDNA sequence of PKM2 (1.6 kb) was inserted to mouse Nphs2 promoter (1.1 kb) constructor. (**B**–**D**) Western blotting of PKM2, PKM1, podocyte marker (nephrin, podocin), and proximal tubule marker AQP1 in glomeruli, tubule, and cortex of PPKM2Tg and WT mice (*n =* 3 per group, *P < 0.05 versus WT). (**B**) Representative image. (**C** and **D**) Quantification of data. (**E**) PK activity in glomeruli, tubule, and cortex of PPKM2Tg and WT mice. (WT [*n =* 4], PPKM2Tg [*n =* 3]; *P < 0.05 versus WT). (**F**) Schema of prevention study designed using STZ-induced diabetic WT (WT 7MSTZ) or PPKM2Tg mice (Tg 7MSTZ). Mice were given 5 consecutive days of i.p. injections of 50 mg streptozotocin (STZ)/kg body weight (50 mg/kg) or vehicle at age of 8 weeks, and they were harvested after 7 months since diabetes onset. (**G**) Fasting blood glucose 7 months after STZ. (**H**) Kidney weight/body weight ratio 7 months after STZ. (**I**) Albumin creatinine ratio 7 months after STZ. Nondiabetic WT mice (*n =* 3); PPKM2Tg mice (*n =* 3); WT 7MSTZ mice (*n =* 6); Tg 7MSTZ mice (*n =* 6). *P < 0.05; **P < 0.01. Data are mean ± SEM, 2-way ANOVA followed by correction for multiple comparison.

To investigate the specific effect of PKM2 activation in podocytes against DN, transgenic mice with PKM2 specifically targeted to the podocyte were generated with mouse podocin promoter (Figure 1A). Analysis of the glomeruli from these mice showed 1.5-fold increase in PKM2 protein levels without compensation of PKM1, compared with WT littermates (Figure 1, B–D). Pyruvate kinase (PK) activity in the glomeruli of PPKM2Tg mice was elevated by 35% (Figure 1E), indicating that the increase of PK activity was due to the elevation of PKM2 levels in the podocyte. In contrast, PKM2 protein expression and activity did not change significantly in renal tubule of PPKM2Tg compared with WT littermates (Figure 1, B, C, and E). Furthermore, PKM1 and PKM2 expression in other tissues of PPKM2Tg mice was not altered (Supplemental Figure 1; supplemental material available online with this article; https://doi.org/10.1172/jci.insight.155260DS1).

### Assessments of diabetes-induced glomerular abnormalities in PPKM2Tg mice.

To evaluate the renal protective role of PKM2 specifically in podocytes, diabetes was induced in PPKM2Tg mice and WT littermates by streptozotocin (STZ) injection to cause insulin deficiency (Figure 1F). After 7 months since diabetes onset, PPKM2Tg mice exhibited lower kidney weight and kidney/body weight ratio versus diabetic WT mice (Supplemental Figure 2C and Figure 1H). Albuminuria and albumin/creatinine ratio (ACR) were significantly attenuated in diabetic PPKM2Tg mice (Supplemental Figure 2D and Figure 1I). Systemic physiological characteristics of PPKM2Tg mice, including fasting blood-glucose levels, body weight, water intake, urine volume, and blood pressure, were not different compared with littermate WT mice in either diabetic or nondiabetic conditions (Figure 1G and Supplemental Figure 2). Body weights of diabetic PPKM2Tg and WT mice were greater than their respective body weights before STZ injection (Supplemental Figure 2G).

**Figure 2:**
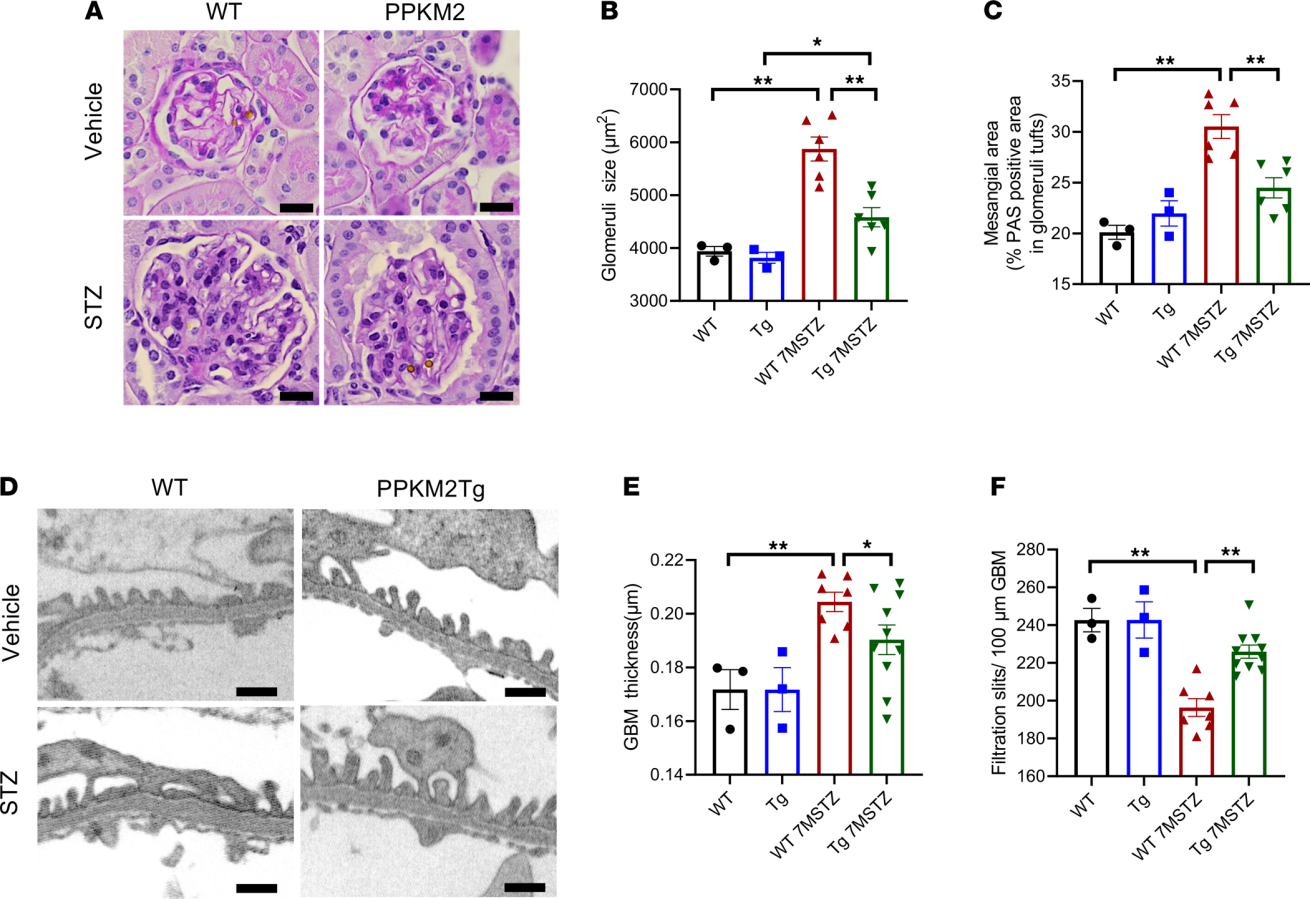
PPKM2Tg prevented diabetes-induced glomerular pathology. (**A**) Representative image of glomeruli 7 months after STZ by PAS staining. *n =* 10–20 images of PAS-stained kidney sections for each mouse. Scale bars: 20 μm. (**B**) Glomerular size 7 months after STZ. (**C**) Mesangial area 7 months after STZ. (**D**) Representative images of glomerular basement membrane (GBM) thickness. Nondiabetic WT mice (*n =* 3); PPKM2Tg mice (*n =* 3); WT 7MSTZ mice (*n =* 6); Tg 7MSTZ mice (*n =* 6). *P < 0.05; **P < 0.01. Scale bars: 0.5 μm. (**E**) Glomerular basement membrane (GBM) thickness 7 months after STZ. Average of 100 measurements for each mouse. (**F**) Slits per 100 μm of GBM. Nondiabetic WT (*n =* 3); nondiabetic PPKM2Tg (*n =* 3); WT 7MSTZ mice (*n =* 7); PPKM2Tg STZ mice (*n =* 10). *P < 0.05, **P < 0.01. Data are mean ± SEM, 2-way ANOVA followed by correction for multiple comparison.

Histological analysis showed that diabetic PPKM2Tg mice had smaller glomeruli size and less mesangial expansion than diabetic WT mice (Figure 2, A–C). Similarly, diabetic PPKM2Tg mice exhibited significantly decreased GBM thickness (Figure 2, D and E). In contrast, the number of filtration slit pores per 100 μm of GBM was increased in diabetic PPKM2Tg mice by 15%, indicating less podocyte foot process effacement compared with diabetic WT mice (Figure 2, D and F). Interestingly, these improvements of glomerular structural abnormalities in diabetic PPKM2Tg mice were not observed until 7 months of diabetes duration. At 4 months of diabetic duration, ACR — but not glomerular pathologies — was improved in PPKM2Tg (Supplemental Figure 3). These data show that PKM2 overexpression specifically in podocytes prevented dysfunction and pathology of the entire glomeruli, independently of systemic hyperglycemia induced by diabetes.

### Gene expression profiles of metabolic, fibrotic, and inflammatory markers in the glomeruli of diabetic PPKM2Tg mice.

**Figure 3:**
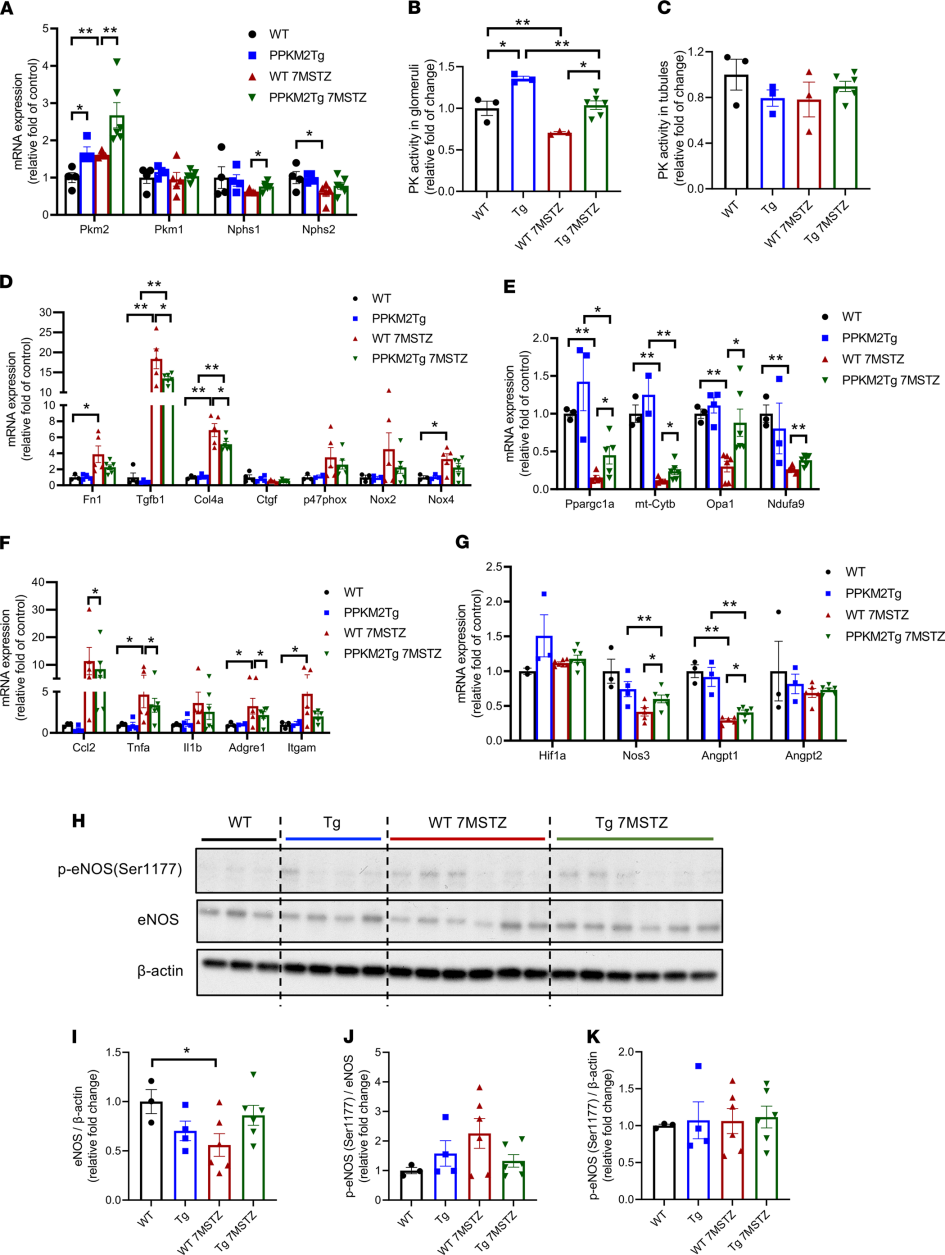
PKM2 overexpression improved glomerular metabolic markers 7 months after STZ. (**A**) mRNA level of PKM1, PKM2, and podocyte markers in the glomeruli of diabetic PPKM2Tg mice 7 months after STZ. (**B**) PK activity of PPKM2Tg glomeruli 7 months after STZ. (**C**) PK activity of PPKM2Tg tubules 7 months after STZ. (**D**) Fibrotic and oxidative stress gene expression in the glomeruli of PPKM2Tg 7 months after STZ. (**E**) mRNA of mitochondrial-related genes in the glomeruli of PPKM2Tg 7 months after STZ. (**F**) mRNA expression of inflammatory genes in the glomeruli of diabetic PPKM2Tg mice. (**G**) mRNA expression of endothelial trophic genes in the glomeruli of diabetic PPKM2Tg mice. WT mice (*n =* 4); PPKM2Tg mice (*n =* 4); WT 7MSTZ mice (*n =* 5); Tg 7MSTZ mice (*n =* 6). *P < 0.05; **P < 0.01. (**H**) Representative Western blotting image of p-eNOS (Ser1177) and total eNOS in glomeruli of diabetic PPKM2Tg versus WT mice. (**I**–**K**) are the quantification data. WT mice (*n =* 3); PPKM2Tg mice (*n =* 5); WT 7MSTZ mice (*n =* 7); Tg 7MSTZ mice (*n =* 7). *P < 0.05. Data are mean ± SEM, 2-way ANOVA followed by correction for multiple comparisons with Tukey’s post hoc test.

*Pkm2* mRNA levels, not *Pkm1*, remained elevated in diabetic PPKM2Tg glomeruli. Expressions of podocyte markers nephrin (*Nphs1*) and podocin (*Nphs2*) were decreased by diabetes but elevated to nondiabetic levels in diabetic PPKM2Tg mice (Figure 3A). Glomerular PK activities of diabetic WT and PPKM2Tg mice were both decreased, although they were still significantly higher in diabetic PPKM2Tg (Figure 3B). Interestingly, PK activities did not change in the tubules of WT and PPKM2Tg mice with or without diabetes (Figure 3C), suggesting the specificity of PKM2 overexpression in PPKM2Tg mice.

Fibrotic genes in the glomeruli were elevated by diabetes, including fibronectin (*Fn1*), TGFβ1 (*Tgfb1*), and collagen α-1 (IV) chain (*Col4a*), with significant attenuation in diabetic PPKM2Tg mice (Figure 3D). Gene profiling of ROS-related enzymes showed that diabetes elevated *p47phox*, *Nox2*, and *Nox4* mRNA levels in WT mice. However, in PPKM2Tg mice, elevations of these ROS-related genes were mitigated compared with diabetes WT mice (Figure 3D), suggesting that lower ambient ROS was generated in the glomeruli by diabetes in PPKM2Tg mice.

Mitochondrial-related genes were studied, showing that systemic PKM2 activation improved mitochondrial function and reduced ROS production (22). Expressions of mitochondrial-related genes *Ppargc1a* (PGC1α), cytochrome b component of complex protein III (*mt-Cytb*), *Opa1*, and *Ndufa9* (a component of complex protein I) were all reduced significantly by diabetes. However, the expressions of these mitochondrial genes were higher in diabetic PPKM2Tg versus WT mice (Figure 3E). Expressions of inflammatory cytokines in the glomeruli were assessed and showed that diabetes elevated the expressions of *Ccl2*, *Tnfa*, *Il1b*, *Adgre1* (*F4/80*), and *Itgam* (*CD11b*) in both PPKM2Tg and WT mice, but they were attenuated in diabetic PPKM2Tg mice (Figure 3F). These data indicate that PPKM2Tg mice prevented mitochondrial damage and ROS accumulation by hyperglycemia.

### Endothelial trophic factors in the glomeruli of diabetic PPKM2Tg mice and patients with diabetes.

An important function of the podocytes is their responses to hypoxia and subsequent secretions of endothelial trophic factors (24). The expressions of hypoxia inducible factor 1α (*Hif1a*), endothelial nitric oxide synthase eNOS (*Nos3*), angiopoietin 1 (*Angpt1*), and angiopoietin 2 (*Angpt2*) were assessed; *Nos3* and *Angpt1* showed a significant decline in diabetic WT mice versus non-DM WT mice. The mRNA expressions of *Nos3* and *Angpt1* also decreased in diabetic PPKM2Tg mice but remained higher than diabetic WT mice (Figure 3G). Protein levels of eNOS in the glomeruli confirmed the mRNA changes (Figure 3, H and I). Interestingly, phosphorylation of eNOS (p-eNOS)/eNOS were increased in the glomeruli of diabetic WT mice versus non-DM WT mice, whereas no changes were detected between DM and non-DM PPKM2Tg mice (Figure 3, H–K).

**Figure 4:**
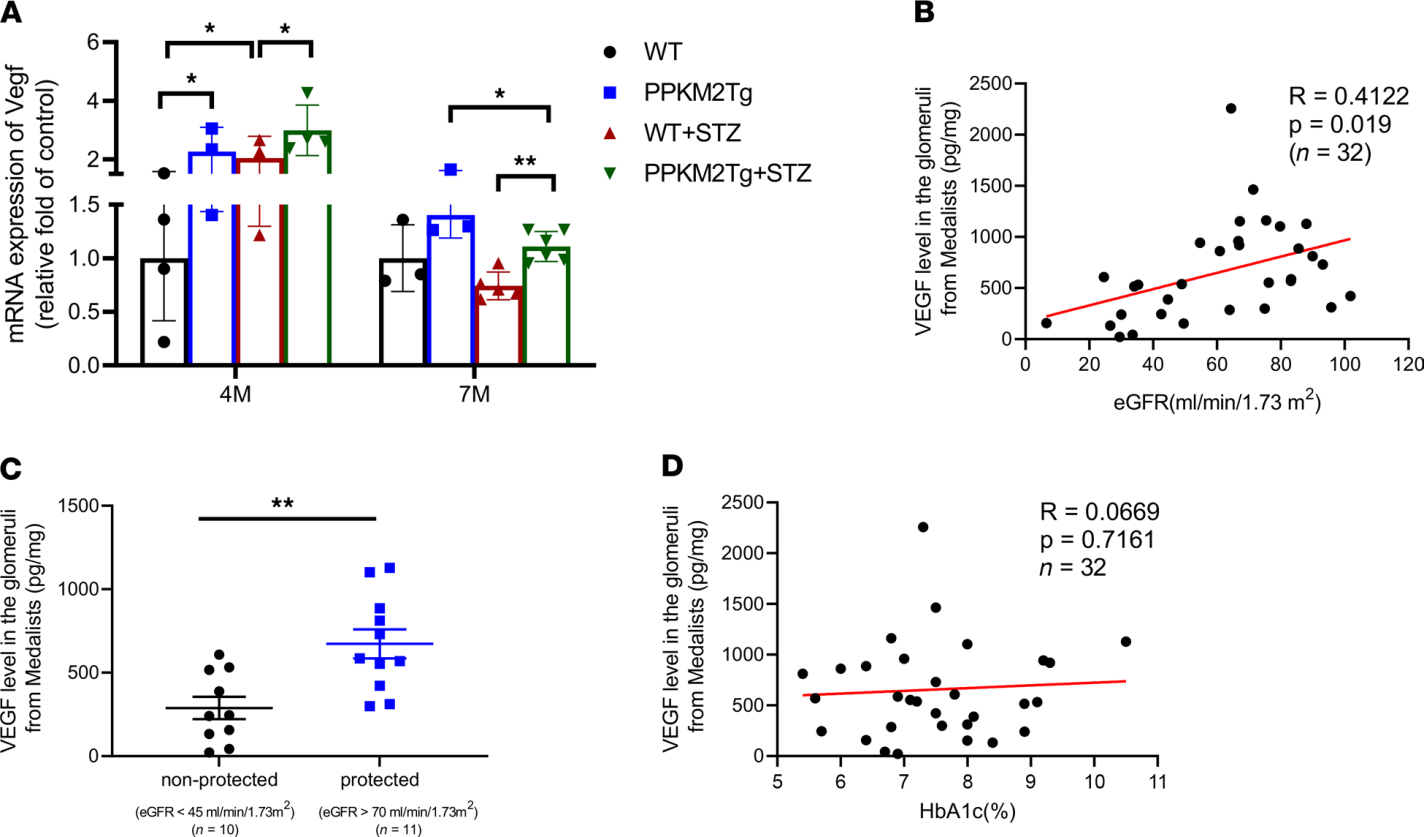
VEGF expression in the glomeruli of diabetic PPKM2Tg mice and Medalist patients. (**A**) VEGF mRNA expression in PPKM2Tg 4 and 7 months after STZ. WT mice (*n =* 4); PPKM2Tg mice (*n =* 4); WT 7MSTZ mice (*n =* 5); Tg 7MSTZ mice (*n =* 6). *P < 0.05; **P < 0.01. Data are mean ± SEM, 2-way ANOVA followed by correction for multiple comparison. (**B**) Pearson correlation of VEGF levels in the glomeruli of Medalists with eGFR levels (*R* = 0.4122, *P =* 0.019, *n =* 32). (**C**) VEGF levels from glomeruli of nonprotected (eGFR < 45 mL/min/1.73 m^2^) (*n =* 10) and protected (eGFR > 70 mL/min/1.73 m^2^) (*n =* 11) individuals from 50 Year Medalist Study. **P < 0.01. (**D**) Pearson correlation of VEGF levels in the glomeruli of Medalists with HbA1c (*R* = 0.0669, *P =* 0.7161, *n =* 32).

*Vegf* mRNA expressions in the glomeruli were elevated in both diabetic WT and PPKM2Tg mice at 4 months since diabetes onset compared with non-DM WT mice, but they were not increased at 7 months since diabetes onset (Figure 4A). Similar to *Vegf* expressions at 4 months since diabetes onset, the glomerular gene expression profiles were not impaired in WT or PPKM2Tg mice (Supplemental Figure 4). VEGF expressions were also studied in the glomeruli of Medalists with T1D of 50 years or longer. Glomeruli were isolated from autopsy kidneys from 32 people with T1D (Table 1). The levels of VEGF in the glomeruli, as measured by ELISA, correlated positively with eGFR as a continuous variable with *R* = 0.4122 and *P =* 0.019 (Figure 4B). Glomerular VEGF levels significantly elevated by 2- to 3-fold in a subset of Medalists protected against CKD (eGFR > 70 mL/min/1.73 m^2^) compared with a subset with lower eGFR (eGFR < 45 mL/min/1.73 m^2^) (Figure 4C and Supplemental Table 1). In addition, VEGF levels in glomeruli did not correlate with HbA1c (Figure 4D). Overall, these data suggest VEGF as a potential protective factor against DN, independent from glycemic control in chronic diabetic conditions.

### Characterization of glycolysis and mitochondrial functions in the glomeruli of PPKM2Tg mice.

**Figure 5:**
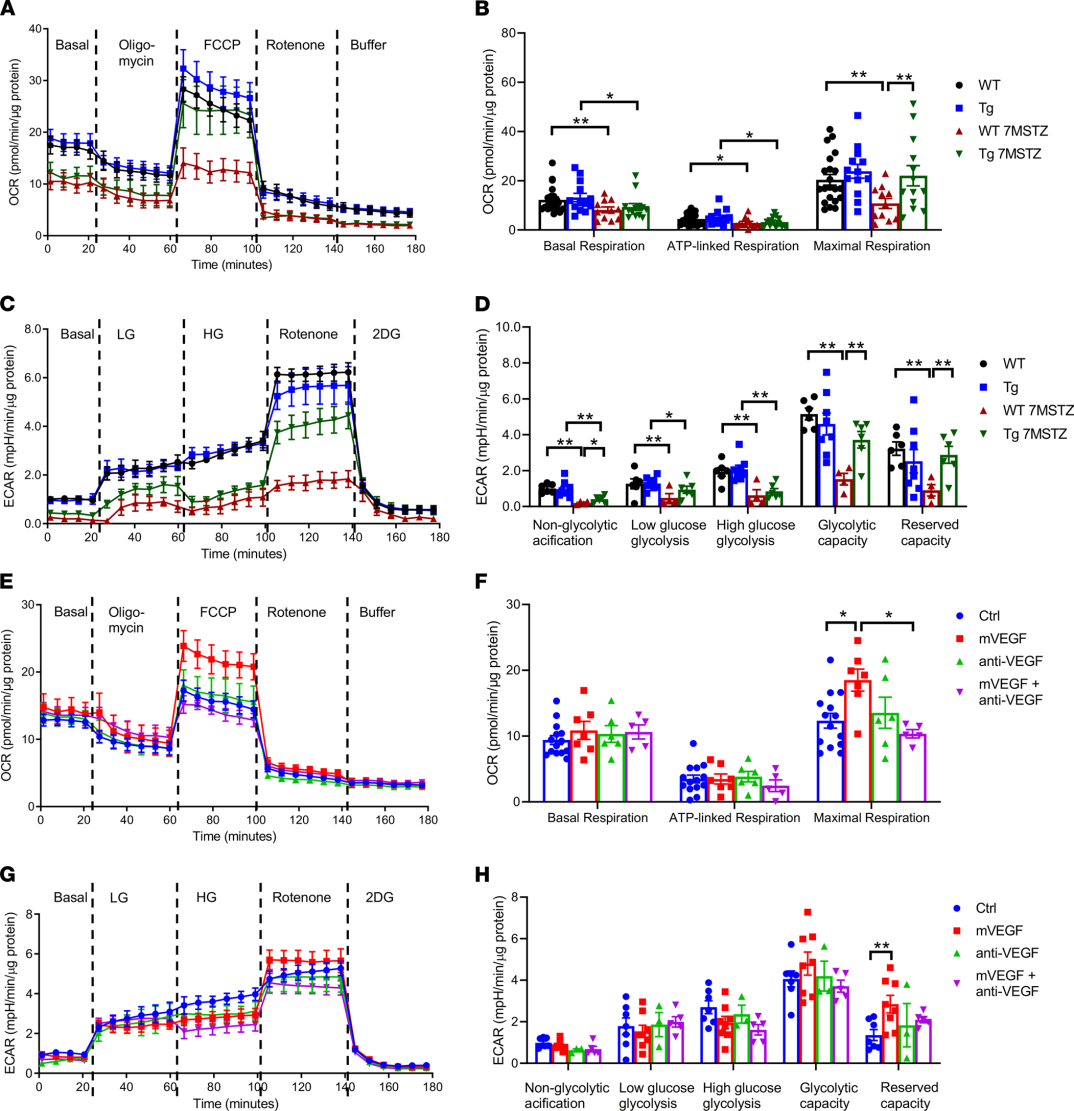
PPKM2Tg mice showed improvement of glomerular mitochondrial function and glycolysis 7 months after STZ. (**A** and **C**) OCR (**A**) and ECAR (**C**) in the glomeruli of WT and PPKM2Tg mice 7 months after STZ were measured by Seahorse. (**B** and **D**) The quantitated data from **A** and **C**. For OCR **A** and **B**, WT mice (*n =* 21); PPKM2Tg mice (*n =* 13); WT 7MSTZ mice (*n =* 11); Tg 7MSTZ mice (*n =* 13). *P < 0.05; **P < 0.01. For ECAR **C** and **D**, WT mice (*n =* 6); PPKM2Tg mice (*n =* 8); WT 7MSTZ mice (*n =* 4); Tg 7MSTZ mice (*n =* 6). *P < 0.05; **P < 0.01. Mouse recombinant VEGF (2 ng/mL), VEGF neutralizing antibody (anti-VEGF) (1 μg/mL), and premix of mVEGF with anti-VEGF were incubated for 2 hours in WT glomeruli. (**E**–**H**) OCR (**E**) and ECAR (**G**) were measured and quantitated data were presented (**F** and **H**). For OCR (**E** and **F**), control group (*n =* 14); mVEGF group (*n =* 7); anti-VEGF group (*n =* 6); mVEGF + anti-VEGF group (*n =* 5). *P < 0.05. For ECAR (**G** and **H**), control group (*n =* 7); mVEGF group (*n =* 8); anti-VEGF group (*n =* 3); mVEGF + anti-VEGF group (*n =* 5). **P < 0.01. Data are mean ± SEM, 2-way ANOVA followed by correction for multiple comparisons with Tukey’s post hoc test.

To investigate whether PPKM2Tg can affect glucose metabolism in whole glomeruli in parallel with improvements of mitochondrial and ROS regulatory pathways, Seahorse assays were performed to assess the metabolic flux in glomeruli. Nondiabetic PPKM2Tg mice did not differ from WT mice in various stages of oxygen consumption rate (OCR). OCR of WT mice after 7 months since diabetes onset was diminished significantly at all stage of respiration rate, including basal to maximal respiration from electron transport chain. However, diabetic PPKM2Tg mice (Tg 7MSTZ) did not exhibit any reduction of OCR in maximal respiration (Figure 5, A and B). Assessments of glycolysis by extracellular acidification rate (ECAR) showed that diabetes significantly decreased all aspects of glucose-induced changes (glucose concentrations: 0 mM, 5 mM, 25 mM) in glycolysis and reserve capacities (Figure 5, C and D). In accordance with OCR, rotenone-induced ECAR in diabetic PPKM2Tg mice was significantly increased, which almost normalized diabetes-induced reductions of glycolysis from mitochondria (Figure 5, C and D). These data suggest that PPKM2Tg mice prevented mitochondrial dysfunction and ROS accumulation related to hyperglycemia and diabetes by improvement of mitochondrial function and glycolytic flux in the whole glomeruli with activation of PKM2 in the podocytes.

### Regulation of mitochondrial function and glycolysis in glomeruli by VEGF.

The overexpression of PKM2 selectively in the podocytes prevented diabetes-induced abnormalities in glycolysis and mitochondrial dysfunction of the whole glomeruli. These findings suggest that metabolites or cytokines from the podocytes were able to normalize other glomerular cells such as endothelial cells in a paracrine manner. We selected to study the role of VEGF as the potential paracrine effector due to findings that diabetic PPKM2Tg mice preserved VEGF expression and functions of eNOS diminished by chronic diabetes (Figure 3, G–I, and Figure 4A). In addition, studies by Eremina et al. have demonstrated the importance of VEGF from podocytes for the maintenance of glomerular structure (15).

The results in Figure 5, E and F, show that incubation of mouse recombinant VEGF (2 ng/mL) could significantly elevate maximal OCR of glomeruli from WT mice. These VEGF effects were inhibited by the addition of anti-VEGF neutralizing antibodies. Interestingly, anti-VEGF alone did not inhibit OCR in WT glomeruli, suggesting that mitochondrial metabolism in the glomeruli was not affected by the loss of VEGF after 2 hours (Figure 5, E and F). This is consistent with the results of the ECAR analysis, which indicated that VEGF elevated glomerular ECAR in the presence of rotenone and that the elevated ECAR was reduced to control levels by addition of anti-VEGF (Figure 5, G and H).

**Figure 6:**
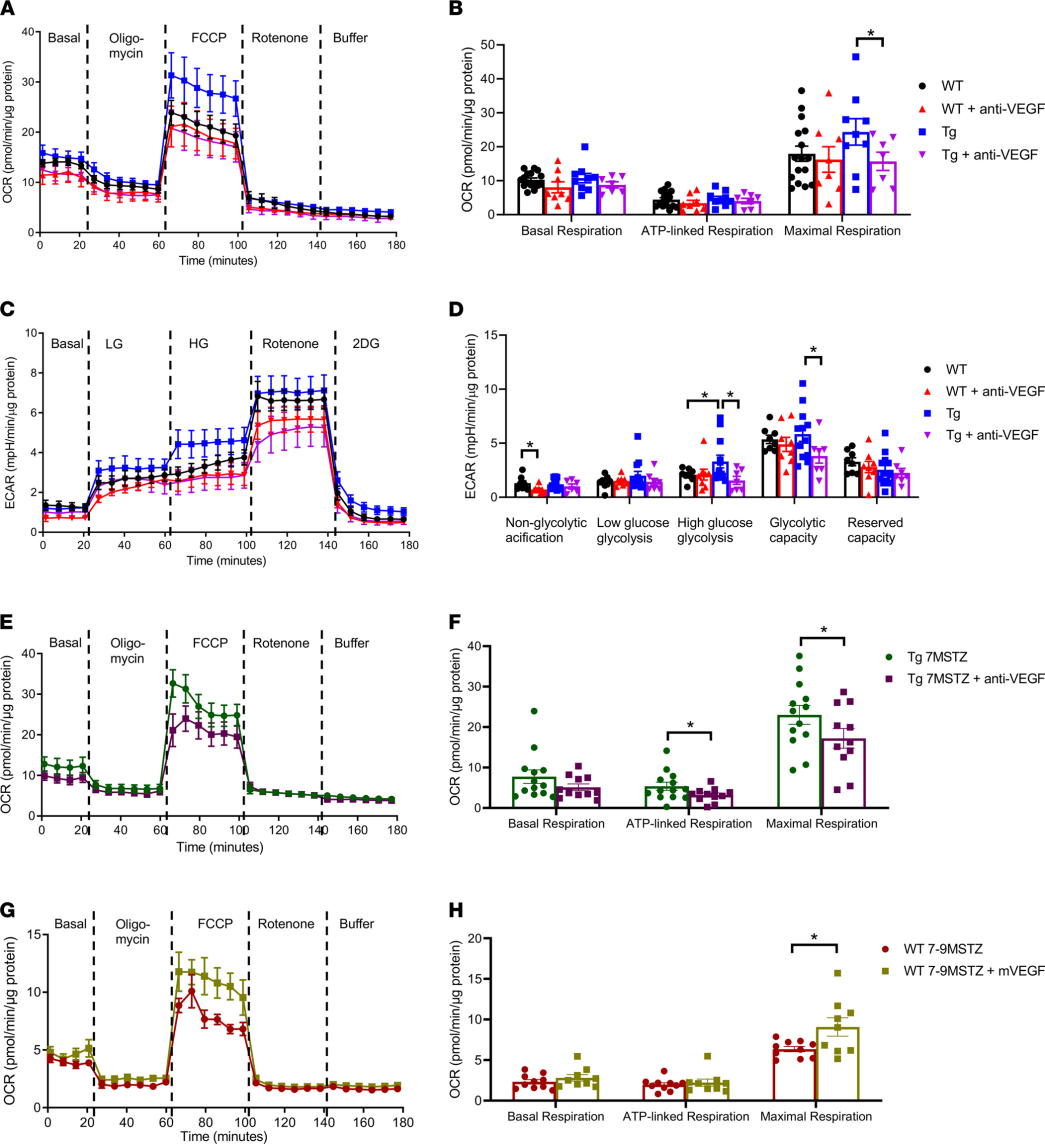
PPKM2Tg regulated VEGF production in the regulation of mitochondrial function in glomeruli. Glomeruli from WT and PPKM2Tg mice were incubated with anti-VEGF(1 μg/mL) for 24 hours, and a Seahorse assay was performed. (**A**–**D**) Representative curve of OCR (**A**) and ECAR (**C**), as well as quantitated data (**B** and **D**), were shown. For OCR, WT (*n =* 16); WT + anti-VEGF (*n =* 7); Tg (*n =* 9); Tg + anti-VEGF (*n =* 7). *P < 0.05. For ECAR, WT (*n =* 8); WT + anti-VEGF (*n =* 8); Tg (*n =* 12); Tg + anti-VEGF (*n =* 7). *P < 0.05. Data are mean ± SEM, 2-way ANOVA followed by correction for multiple comparisons with Tukey’s post hoc test. Glomeruli from diabetic PPKM2Tg mice were treated with anti-VEGF (10 μg/mL) for 24 hours. (**E** and **F**) Representative curve of OCR and quantitated data were shown in **E** and **F**. Tg 7MSTZ (*n =* 13); Tg 7MSTZ + anti-VEGF (*n =* 11). *P < 0.05. Diabetic WT mice 7–9 months after STZ (7-9MSTZ) were incubated with mVEGF (100 ng/mL) for 24 hours and addition of mVEGF (100 ng/mL) 1 hour before Seahorse assay. (**G** and **H**) Representative curve of OCR and quantitated data. WT 7-9MSTZ (*n =* 10); WT 7-9MSTZ + VEGF 24/1h (*n =* 9). *P < 0.05. Data are mean ± SEM.

To determine the attribution of elevated VEGF on the enhancement of mitochondrial functions in the glomeruli of PPKM2Tg mice, glomeruli were treated with anti-VEGF for 24 hours. Maximum respiration rate of OCR in PPKM2Tg glomeruli was significantly reduced by anti-VEGF treatment but was ineffective in WT glomeruli (Figure 6, A and B). The glycolytic rate of glomeruli from PPKM2Tg mice in high glucose conditions and the glycolytic capacity were significantly elevated compared with WT mice, and they were all inhibited by anti-VEGF (Figure 6, C and D). Similar suppressive effects on mitochondrial respiration and glycolysis by anti-VEGF were observed in PPKM2Tg mice after 7 months since diabetes onset, with partial declines of OCR and ECAR compared with diabetic WT (Figure 6, E and F, and Supplemental Figure 5, A and B). These results suggest that VEGF not only regulated mitochondrial function and glycolysis in glomerular endothelial cells in nondiabetic conditions, but it also regulated mitochondrial function and glycolysis of glomerular endothelial cells in the nondiabetic condition, but also in DN. Additionally, pretreatment of VEGF for 24 hours (100 ng/mL) and addition of VEGF (100 ng/mL) 1 hour before assay partially reversed glomerular mitochondrial dysfunction due to metabolic memory after 7–9 months since diabetes onset (Figure 6, G and H, and Supplemental Figure 5, C and D). These ex vivo studies of glomeruli as a whole could provide information on intercellular interactions. However, studying effects of VEGF on individual cell types is also important. Since VEGF mainly affects endothelial cells in the glomeruli, we tested VEGF effects on primary cultured mouse glomerular endothelial cells. Consistent with ex vivo Seahorse experiments, VEGF also activated OCR in maximal respiration rate in the primary endothelial cells (Supplemental Figure 6, A–D). Collectively, these data support that enhanced VEGF expression in PPKM2Tg mice can improve mitochondrial metabolism of other glomerular cells such as endothelial cells, even with exposure to diabetes of a long duration.

### PKM2 and its enzymatic activity in the regulation of VEGF expression in podocytes.

**Figure 7:**
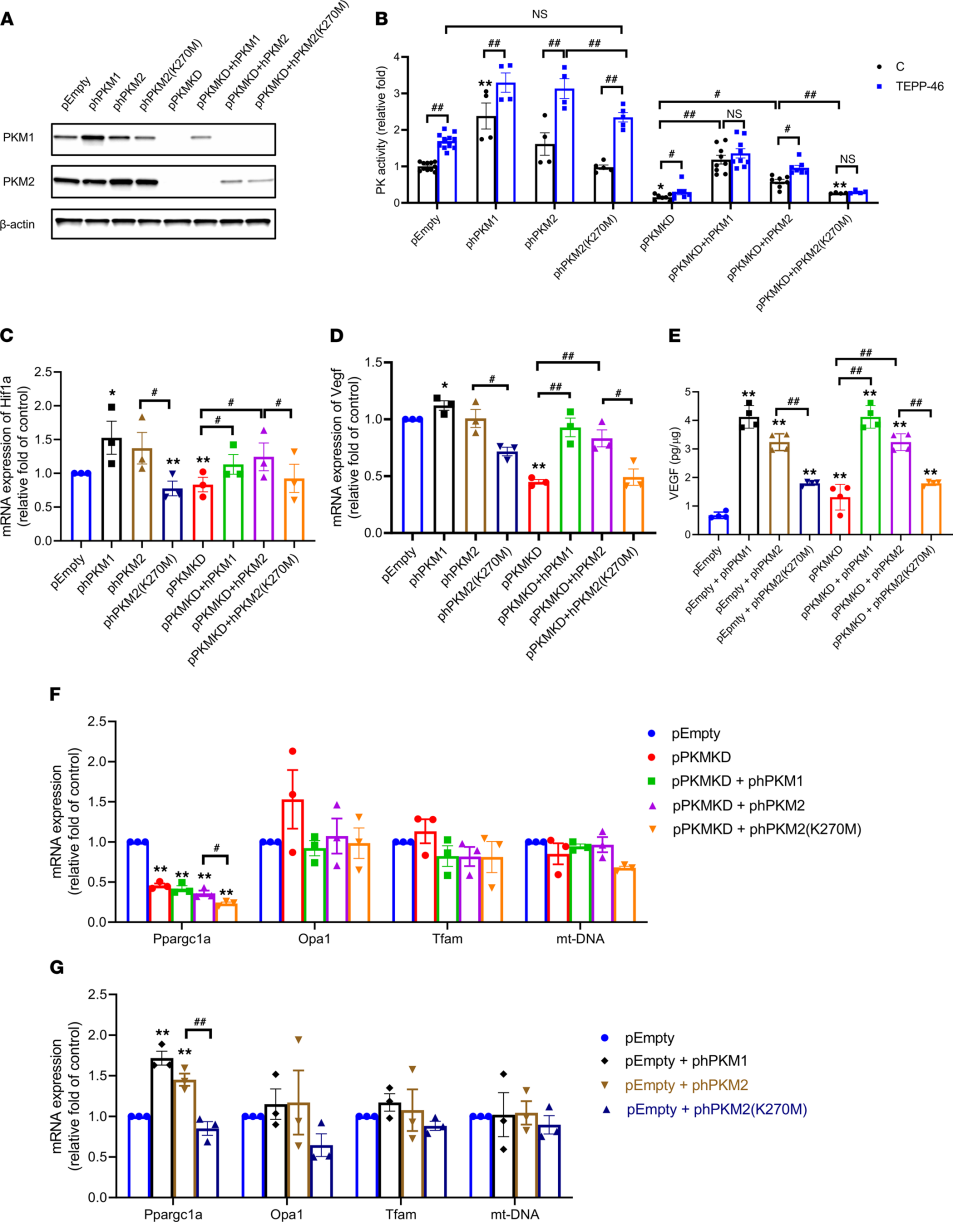
PKM2 and its enzymatic activity in regulation of VEGF production in podocyte. (**A**) Western blotting of PKM1 and PKM2 in pEmpty, phPKM1, phPKM2, phPKM2(K270M), pPKMKD, pPKMKD + phPKM1, pPKMKD + phPKM2, and pPKMKD + phPKM2(K270M) podocyte stable cell lines. (**B**) PK activity of pEmpty (*n =* 11), phPKM1 (*n =* 4), phPKM2 (*n =* 4), phPKM2(K270M) (*n =* 5), pPKMKD (*n =* 7), pPKMKD + phPKM1 (*n =* 9), pPKMKD + phPKM2 (*n =* 7), and pPKMKD + phPKM2(K270M) (*n =* 4) podocyte stable cell lines in basal level and 10 μM TEPP-46 treatment. *P < 0.05, **P < 0.01 versus pEmpty; ^#^*P <* 0.05, ^##^*P <* 0.01 for each pair of the compared groups. (**C** and **D**) mRNA expression of *Hif1a* and *Vegf* in pEmpty, phPKM1, phPKM2, phPKM2(K270M), pPKMKD, pPKMKD + phPKM1, pPKMKD + phPKM2, and pPKMKD + phPKM2(K270M) lines. *n =* 3; *P < 0.05, **P < 0.01. ^#^*P* < 0.05, ^##^*P* < 0.01 for each pair of the compared groups. (**E**) VEGF levels 24 hours in culture media of pEmpty, phPKM1, phPKM2, phPKM2(K270M), pPKMKD, pPKMKD + phPKM1, pPKMKD + phPKM2, and pPKMKD + phPKM2(K270M) lines. *n =* 4; **P < 0.01 versus pEmpty; ^##^*P <* 0.01 for each pair of the compared groups. (**F**) mRNA expression of mitochondrial-related genes in pEmpty, pPKMKD, pPKMKD + phPKM1, pPKMKD + phPKM2, and pPKMKD + phPKM2(K270M) lines. (**G**) mRNA expression of mitochondrial-related genes in pEmpty, phPKM1, phPKM2, and phPKM2(K270M) lines. *n =* 3; **P < 0.01 versus pEmpty; ^#^*P <* 0.05, ^##^*P <* 0.01. Data are mean ± SEM, 2-way ANOVA followed by correction for multiple comparisons with Tukey’s post hoc test.

Previously, it was reported that PKM2, an allosteric enzyme, can mediate its actions either through its enzymatic activities or by transport to the nucleus, where it transforms into monodimeric form and then aggregates to tetrameric form (25–32). Its tetrameric form has been found to mediate changes in cellular apoptosis and proliferation independently from its enzymatic activity (28, 31, 32). To investigate whether PKM2’s enzymatic activities are essential for the regulation of VEGF expression in podocyte, we generated several podocyte stable cell lines by knocking down endogenic murine PKM1/2 and then overexpressing human PKM1/2 WT and mutated enzymatically inactive isoforms. We knocked down endogenic murine PKM1 and PKM2 by lentiviral shRNA to generate the PKM–double knockdown stable cell line (pPKMKD) and empty cell line (pEmpty). Then, pEmpty and pPKMKD cells were overexpressed or reexpressed with human PKM1 (pEmpty + phPKM1, pPKMKD + phPKM1), PKM2 (pEmpty + phPKM2, pPKMKD + phPKM2), or enzymatically inactive PKM2 (pEmpty + phPKM2(K270M), pPKMKD + phPKM2[K270M]) to study their function, respectively in the podocyte. PKM2(K270M) was generated with a mutation at lysine(AAA) and mutated to methionine (ATG), which still can tetramerize but lack PK activity, to evaluate enzymatic activity of PKM2 on podocyte metabolism (33). The protein levels of PKM1 and PKM2 in these cells were evaluated (Figure 7A), with over 90% knockdown of PK activity in the pPKMKD line and with recovery or increase of PK activity by phPKM1 and phPKM2 but not phPKM2(K270M; Figure 7B). The addition of small molecule TEPP-46, which activated PKM2 enzymatic activity by increasing tetramerization, further increased PK activity in all the lines, except pPKMKD + phPKM2(K270M) or pPKMKD + phPKM1 cells (Figure 7B).

Interestingly, knocking down PKMs in podocyte decreased *Hif1a* and, subsequently, dramatically decreased *Vegf* expression. The expressions of *Hif1a* and *Vegf* were normalized by the reexpression of phPKM1/2, but not enzymatically inactive phPKM2(K270M) (Figure 7, C and D). Overexpression of hPKM1/2 modestly increased *Hif1a/Vegf* mRNA expression and was suppressed by pEmpty + phPKM2(K270M), suggesting aggregation of phPKM2(K270M) with endogenous PKM2 (Figure 7, C and D). Similar results were observed by ELISA, and the levels of VEGF in culture medium were increased by reexpression or overexpression of PKM1/2; however these accumulations were suppressed by phPKM2(K270M) compared with phPKM2 cells (Figure 7E). These data suggest that PKM1/2 regulated the HIF1α/VEGF axis in the podocyte and VEGF secretion via activation of mitochondrial flux. In addition, enzymatic activity of PKM2, which exerted its action as a glycolytic enzyme to convert phosphoenolpyruvate and ADP to pyruvate and ATP, was necessary for mediating this process.

To further investigate PKM2 and its enzymatic activity on the regulation of podocyte fuel metabolism, mitochondrial-related genes were determined. Mitochondrial DNA (mtDNA), *Opa1*, and mitochondrial transcription factor A (*Tfam*) were not significantly altered by PKM overexpression, knockdown, or reexpression; only *Ppargc1a* responded to PKM expression and activities (Figure 7, F and G). Overexpression of phPKM1 or phPKM2 induced *Ppargc1a* expression but not with phPKM2(K270M), and its expression was reduced in pPKMKD cells. However, reexpression of hPKM1/2 did not rescue the expression of *Ppargc1a* (Figure 7F).

**Figure 8:**
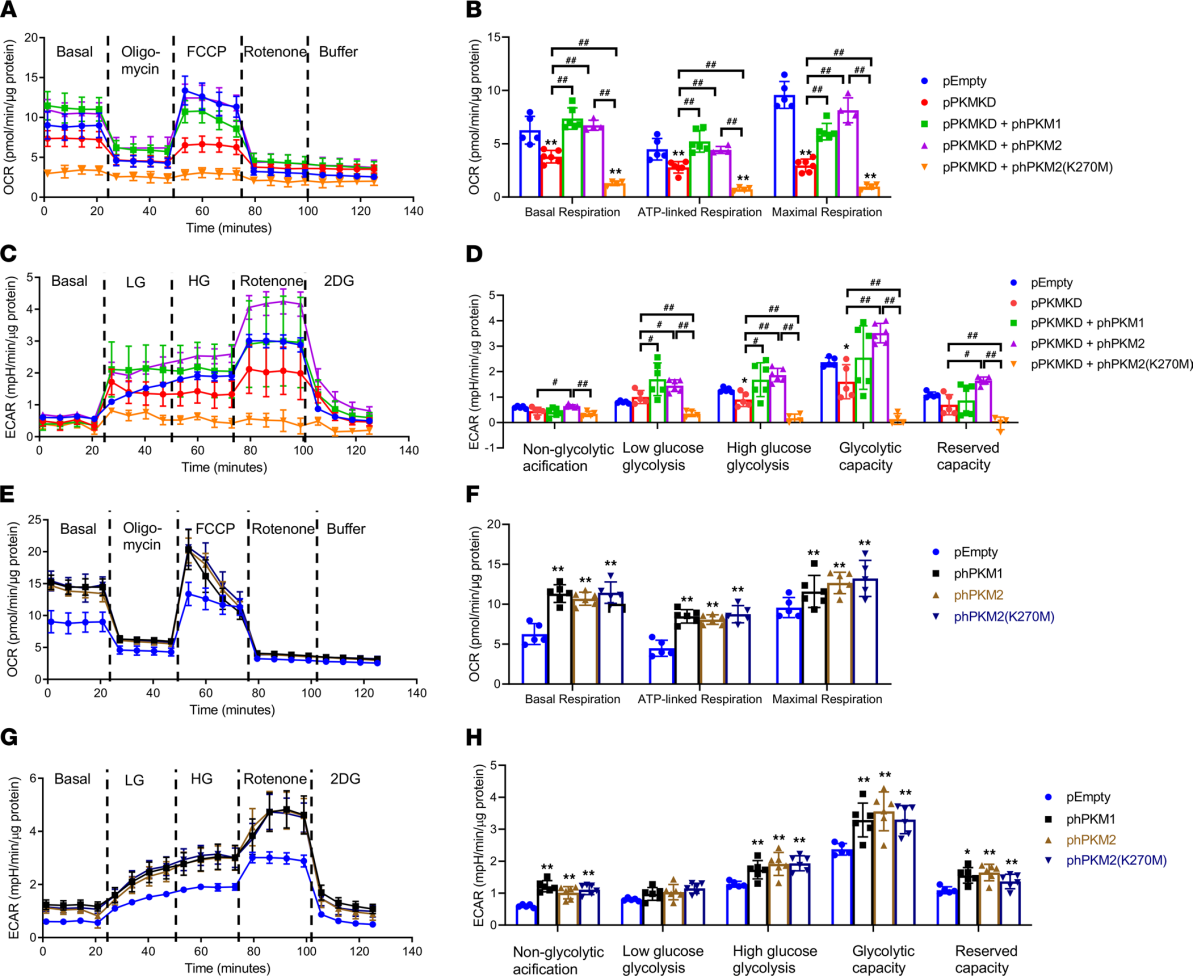
PKM2 and its enzymatic activity in regulation of mitochondrial function and glycolysis in podocyte. (**A** and **B**) Representative curve and quantitated data of OCR in pEmpty (*n =* 5), pPKMKD (*n =* 6), pPKMKD + phPKM1 (*n =* 6), pPKMKD + phPKM2 (*n =* 4), and pPKMKD + phPKM2(K270M) (*n =* 4) lines. **P < 0.01 versus pEmpty; ^##^*P <* 0.01 for each pair of the compared groups. (**C** and **D**) Representative curve and quantitated data of and ECAR in pEmpty (*n =* 5), pPKMKD (*n =* 5), pPKMKD + phPKM1 (*n =* 6), pPKMKD + phPKM2 (*n =* 6), and pPKMKD + phPKM2(K270M) (*n =* 4) lines. *P < 0.05 versus pEmpty; ^#^*P <* 0.05, ^##^*P <* 0.01. (**E** and **F**) Representative curve and quantitated data of OCR in pEmpty (*n =* 5), phPKM1 (*n =* 6), phPKM2 (*n =* 6), and phPKM2(K270M) (*n =* 5) lines. **P < 0.01 versus pEmpty. (**G** and **H**) Representative curve and quantitated data of and ECAR in pEmpty (*n =* 5), phPKM1 (*n =* 6), phPKM2 (*n =* 6), and phPKM2(K270M) (*n =* 6) lines. *P < 0.05, **P < 0.01 versus pEmpty. Data are mean ± SD, 2-way ANOVA followed by correction for multiple comparison.

Mitochondrial function and glycolytic flux in podocyte were also studied in these cells. pPKMKD cells exhibited significant lower mitochondrial respiration and glycolytic rate at all stages measured by OCR and ECAR (Figure 8, A–D). These impairments of OCR and ECAR were normalized by reexpression of PKM1/2 but not PKM2(K270M), indicating that PK activity was critical for maintaining podocyte mitochondrial function and glycolysis (Figure 8, A–D). These changes in podocyte metabolism were not directly mediated by PKM’s regulation on mitochondrial-related genes, but rather on glycolytic flux into TCA cycle. Similarly, overexpression of phPKM1/2 further increased mitochondrial function and glycolytic rates with increased mitochondrial biogenesis (Figure 8, E–H). Different from reexpressing cells, pEmpty + phPKM2(K270M) with aggregation of phPKM2(K270M) and endogenous PKM2 also increased mitochondrial fuel metabolism. Collectively, these data suggest that PKM1/2 and its enzymatic activity were necessary for podocyte mitochondrial function and glycolysis by maintaining metabolites needed for mitochondrial function.

## Discussion

In this study, we demonstrate that overexpression of PKM2 targeted to podocytes can prevent multiple molecular, metabolic functional, and pathological changes induced by diabetes in the whole glomeruli. These changes included gene expressions that regulate fibrosis, inflammation, trophic factors for the endothelium, basement membrane, and oxidative stress. Our findings show that targeting PKM2 and glucose metabolism in the podocyte selectively could normalize glycolytic and mitochondrial metabolism of the whole glomeruli, even in the presence of chronic diabetes and hyperglycemia. Furthermore, PKM2 activation in the podocytes improved VEGF expression and normalized glomerular functions and metabolism. These effects required PKM2 with tetrameric structure and glycolytic enzymatic activities.

We made the surprising finding that the activation of 1 glycolytic and allosteric enzyme, PKM2 in the podocytes, can normalize function and metabolism of the entire glomeruli even with 7 months since diabetes onset. Since the high transgene expression of many transgenic models could be detrimental and may not be physiologically appropriate, we generated PPKM2Tg mice using a 1.1 kb podocin promoter, instead of a full-length 2.5 kb podocin promoter, to reduce overactivation of PKM2 expressions in the mice. The size and pathology of PPKM2Tg were investigated until the age of 36 weeks, and no impairment of the development of kidney was observed with the moderate elevation of PKM2 expression in the podocyte. PPKM2Tg mice only exhibited 50% and 35% increases in PKM2 protein and PK activities, respectively, in the glomeruli, and they were similar to the elevation in people protected from DN yet were able to neutralize the adverse effects of chronic hyperglycemia in diabetes. After 7 months since diabetes onset, though the mRNA of PKM2 was increased, the total PK activities in the diabetic glomeruli were significantly decreased by 30% and were normalized in PPKM2Tg mice with improved DN. These results were similarly found in our previous study that mRNA and protein levels of PKM2 were not decreased in diabetic mice models, while its activity and tetramerization were decreased by diabetes-induced oxidation of PKM2 (22). Thus, we suggest that the elevations of PKM2 proteins and activities need to occur to delay the progression of DN. These findings strongly support the importance of podocytes in regulating glomeruli metabolism and functions, likely in a paracrine manner.

The increase of PKM2 activities in the podocyte had differential effects on the structures and functions of glomeruli. Activations of PKM2 in the podocyte did not mitigate all the diabetes-induced changes in the glomeruli. Diabetes-induced glomerular abnormalities, such as oxidative stress markers, mitochondrial genes, and mitochondrial functions, were normalized at both 4 and 7 months after diabetes. Fibrosis was improved by counting the percentage of mesangial area and thickness of GBM, indicating the ECM produced from mesangial cells in PPKM2Tg mice were suppressed after 7 months since diabetes onset. However, the fibrotic genes *Tgfb1* and *Col4a* were significantly reduced yet limited in diabetic PPKM2Tg mice. There is a large number of studies showing that the content of ECM was mediated by both synthesis and degradation (34, 35). Our measurement of these fibrotic factors are on mRNA levels, indicating that the improvement of fibrosis could also be on the regulation of ECM degradation rather than synthesis. Another possibility is that there could be other factors that regulated the fibrosis during this process. Our data show that PKM2 activation in the podocyte has limited protective effect on inflammation and fibrosis but that it strongly normalized mitochondrial and glycolytic abnormalities of the glomeruli, suggesting that, in this model, the pathology of DN could be improved by metabolism even with the presence of some extent of inflammation. Interestingly, the expressions of VEGF, eNOS, and other endothelial trophic genes were initially elevated in 4 months of diabetes and then dramatically decreased at 7 months. The decrease was prevented by the improvement of mitochondrial function in the podocytes due to PKM2 activation. However, PKM2 activation did not prevent the enlargements of glomeruli and kidney sizes, although glomerular function ACR was clearly improved. These findings suggest that increases in kidney size and glomerular diameter might not be detrimental to renal functions or lead to CKD unless their sizes were reduced by chronic hyperglycemia. It is possible that enlargement of the kidney and glomeruli may even have protective functions due to preservation for metabolizing high glucose flux through the glycolysis and mitochondrial pathway.

In parallel with the metabolic changes, the initial elevation of VEGF expressions in the glomeruli at 4 months corresponded with the enlargement of glomeruli size, and subsequently, its reduction corresponded with the changes in glomerular and renal sizes, as observed in advanced diabetic rodent models and patients (18, 36–38). The initial elevation of VEGF in the diabetic glomeruli was not affected by PKM2 overexpression in the podocyte, and its expression was preserved in PPKM2Tg mice after 7 months since diabetes onset. The importance of persistent elevation of VEGF in the glomeruli is also supported by the findings of people from the Medalist Study — which showed that VEGF levels correlated positively with eGFR — whose renal functions were preserved over 50 years duration of T1D. This is the first human diabetes study to our knowledge that measured VEGF expression specifically in the glomeruli and correlated with kidney dysfunction during chronic diabetes but did not correlate with glycemic control. These data from patients strongly suggest that VEGF as a local potential protective factor against DN, even in the presence of hyperglycemia. The importance of paracrine functions of podocytes on glycolysis and mitochondrial function in the glomeruli was clearly demonstrated by Seahorse analysis. We think it would be important to study both glomeruli as a whole and separated single cells. However, VEGF was reported to have very limited effects on the podocyte itself. VEGF produced by the podocyte predominantly exerted its actions by paracrine — but not autocrine — VEGF/VEGFR-2 signaling to the podocyte and regulated the structure and function of endothelial cells (39). Thus, we performed a Seahorse assay only on the whole glomeruli and primary glomerular endothelial cells, and we found that enhanced VEGF expression could improve mitochondrial metabolism of other glomerular cells such as endothelial cells, even with exposure to diabetes of long duration.

It has been reported that, in an unstressed state, podocyte mitochondrial metabolism is not critical for maintaining its function (40). However, in a stress state with glomerular hyperfiltration, such as DN, our data suggest that mitochondrial metabolism in podocytes might be important to maintain glomerular metabolism and function. Furthermore, selective PKM2 activation in the podocytes was able to prevent mitochondrial dysfunction in the entire glomeruli even after 7 months of sever hyperglycemia and diabetes, even though podocyte constituted only 20%–30% of the glomerular cells. To determine the mechanisms of PKM2 activation in podocytes on the whole glomerular glucose metabolism, we focused on the regulation of VEGF expressions, mainly produced in the glomeruli by the podocyte (41, 42). Various growth factors, cytokines, and metabolites secreted from glomerular cells have been reported to contribute to DN in a paracrine manner. Among them, VEGF was one of the most well studied that showed critical actions on glomerular functions (39, 42). Quaggin’s lab has shown that VEGF is required for maintaining glomeruli structure, especially the development of glomerular capillaries and endothelial cell fenestration (15, 43). Specific deletion of VEGF expression in podocytes resulted in failure in the formation of filtration barriers and endothelial cell fenestration (15). Furthermore, declining VEGF signaling was associated with aging across multiple organs, and mild increase of systemic VEGF levels could extend life spans by preventing age-associated capillary loss (44). Our findings confirm that VEGF expression was increased in early stages of DN in concordance to the growth and proliferation of mesangial and endothelial cells. However, its expressions declined in chronic DN in parallel with podocyte loss and endothelial cell dysfunction (45–47). Results from the PPKM2Tg mice showed that PKM2 activation would preserve VEGF, eNOS, and angpt1 expression even with diabetes, and this correlated with the retained eGFR in people protected from CKD with very long duration (>50 years) of T1D. These data indicate that maintenance of VEGF expression could also be a potential protective factor against DN.

It has been reported that VEGF can protect microvasculature injury in DN, but the mechanism is unclear (16). Our findings indicate that PKM2 activation in the podocytes preserves mitochondrial flux and function initially in the podocytes, and then the whole glomeruli, by enhancing and maintaining the VEGF expressions. The preservation of VEGF physiological levels in the glomeruli can enhance glucose’s oxidative phosphorylation in the endothelial cells against hyperglycemia. This hypothesis is supported further by anti-VEGF treatment, which reduced OCR from glomeruli in diabetic PPKM2Tg mice. In addition, exogenic VEGF also elevated oxidative phosphorylation of glomeruli from WT mice, similar to those observed in PPKM2Tg mice. In the presence of chronic diabetes, anti-VEGF reduced mitochondrial metabolism of glucose in both diabetic WT and PPKM2Tg mice, indicating that VEGF has an important role in maintaining mitochondrial function in a diabetic state. However, it is interesting that the glomeruli from nondiabetic WT mice did not respond to anti-VEGF, suggesting that VEGF has a lesser role in maintaining mitochondrial homeostasis in an unstressed condition. These data suggest that VEGF is critically involved in preserving glomerular endothelial mitochondrial functions in the diabetic and other stress states. Previously, VEGF has been shown to stimulate mitochondrial biogenesis in endothelial cells (48). Depletion of VEGF from the endothelium resulted in mitochondrial fragmentation and suppression of glucose metabolism with decreased glucose uptake, lactate production, and mitochondrial oxygen consumption (49). VEGF also activated OCR in maximal respiration rate in the primary endothelial cells. These findings indicate that elevation of glycolysis and mitochondrial metabolism in podocytes would elevate or maintain VEGF expression even in diabetic status. The elevation of VEGF in the glomeruli observed in early stages of DN could be protective against the mitochondrial dysfunction induced by hyperglycemia. This suggestion is supported by a previous report that reduction of VEGF in early stages of STZ-induced DN mice accelerated endothelial and glomerular injuries (16). On the other hand, others have reported that continuous VEGF overexpression in the podocyte led to glomerulopathy (15), proteinuria, glomerulomegaly, GBM thickening, and mesangial expansion (50). These different conclusions of VEGF overexpression in the glomeruli have led to the suggestions that VEGF may have different actions on glomeruli (38, 51). However, our results suggest that podocyte VEGF expression and function are metabolically regulated in response to changes of fuel availability and utilization by various stimuli, such as stress. The continuous or deficient expression of VEGF can cause both metabolic and pathological dysfunctions in the glomeruli (52). Many hormonal and cytokine disorders have displayed similar patterns: for example, hyperinsulinemia with insulin resistance and deficiency in autoimmune diabetes can accelerate atherosclerosis (53–55), and either loss or chronic excessive production of cytokines can contribute to organ dysfunction (37, 56, 57).

At the molecular level, our finding using various deletion and overexpression of PKM2 have strongly demonstrated its regulation on mitochondrial metabolism and podocyte functions. In podocytes, we found that PKM2 and its enzymatic activity were both necessary for maintaining podocyte mitochondrial function and the secretion of VEGF. PKM2 has been reported to mediate multiple functions by either tetramer or dimer forms as a transcription factor (25–32). Its tetrameric form was reported to function independently from its enzymatic function. For example, in endothelial cells, PKM2 regulates cellular proliferation and barrier function independently of its enzymatic activity (31), and others have reported that its tetramer form could bind to the outer membrane of mitochondria and be involved in cell apoptosis (58). Unlike PKM1, which only exist as tetramer, PKM2 as a dimer could also be transported into nuclear and mediated HIF1α and inflammation (28–30). Our results clearly show that PKM2 and its enzymatic activity were both critical for maintaining podocyte mitochondrial function and VEGF secretion, as PKM1 and PKM2 both can affect HIF1α/VEGF expression — but not with PKM2 lacking enzymatic activity. Many studies have showed that mitochondria activities can regulate the activation of HIF1α and VEGF (59); further studies will be needed to determine the mechanism by which PKM2 activation induces the HIF1α/VEGF pathway in podocytes. It is likely that metabolites of the TCA cycle could be involved, since succinate has been reported to be an activator of HIF1α/VEGF cascade (60).

In summary, these findings show that activation or elevation of PKM2 in the podocyte can regulate mitochondrial metabolism of the entire glomeruli. Furthermore, PKM2 activation can prevent mitochondrial dysfunction induced by chronic diabetes and even metabolic memory. Mechanistically, PKM2 enzymatic activities are necessary to mediate glomerular metabolism, partially by maintaining VEGF expression, which is critical for endothelial cells and podocyte function. Thus, PKM2 activation in podocytes acts as a critical role and potential therapeutic target to protect against DN.

## Methods

### Plasmid construction.

Mouse full-length PKM2 cDNA including a BamH1 cutting site is amplified from pLHCX-FLAG-mPKM2 plasmid by PCR. pLHCX-FLAG-mPKM2 was a gift from Lewis Cantley and Matthew Vander Heiden (Addgene plasmid 44239; http://n2t.net/addgene:44239; RRID: Addgene_44239). PCR products were extracted by QIAamp DNA Micro Kit (Qiagen, 56304). Then, PCR products were ligated with pGEMT-Easy vector and transformed in NEB 5-α Competent *E. coli*. After amplification, the plasmid and podocin-promoter vector were digested with BamH1. Both digested DNA were extracted by purification kit. Next, purified mPKM2 transgene was cloned into the BamH1 site of podocin-promoter Gluc-ON reporter vector designed and purchased from GeneCopoeia (MPRM15744-PM02) and transformed in NEB 5-α Competent *E. coli*. After amplification, podocin-promoter PKM2 transgene was extracted by QIAprep Spin Mini prep kit (Qiagen, 27106X4). All constructs were DNA sequenced to assure amplification fidelity.

### Generation of transgenic animal.

Purified transgene constructs were microinjected into C57BL/6 egg pronuclei to generate PPKM2Tg mice. This procedure was performed by Dana-Farber/Harvard Cancer Center Transgenic Mouse Core. Mice were genotyped by Forward: 5′-GCATCCTGTCTATTTTAGAGGTAT-3′, Reverse: 5′-ATTCCAGACTTAATCATCTCCTT-3′.

### Mouse studies.

PPKM2Tg mice were backcrossed with C57BL/6J mice, purchased from The Jackson Laboratory. Male mice were used in mouse studies, and experiments of PPKM2Tg mice were performed with littermate controls. In STZ-induced diabetic mouse studies, male mice at 8 weeks old were injected with either saline or STZ (Sigma-Aldrich) at 50 mg/kg body weight for 5 consecutive days by i.p. injections, and they were maintained with sustained glucose > 400 mg/dL for 7 months in the study.

### Mouse metabolic measurements.

Blood glucose levels were measured from tail-vein blood using contour (Bayer). Urine was collected from 24-hour metabolic caging. Albuminuria was quantified by ACR using the Albuwell M Albumin ELISA kit and the Urinary Creatinine Detection Kit (Arbor Assays).

### Human glomeruli isolation from Medalist Study.

Human glomeruli isolation was performed as described previously (22). Briefly, cortex from human kidneys was dissected and minced on ice. The mixture then passed through 263, 212, 89 μm sieves, and it was washed by cold PBS. The glomeruli were remained on the 89 μm sieve, and they were washed and collected as pellets for studies. Thirty-two samples of glomeruli from Medalists were isolated for experiments in this study.

### Mouse glomeruli isolation.

PBS with Dynabeads M-450 Tosylactivated (Invitrogen, 14013) was perfused into the mouse aorta; kidney tissues were dissected, minced, and digested in 1 mg/mL collagenase A and 100 U/mL DNase I for 30 minutes at 37°C. Digested mixture was then passed through a 100 μm cell strainer and placed on a magnet for purification. The purity of glomeruli was more than 95% confirmed by microscopy.

### Mouse kidney pathology assessment.

Mouse kidneys were fixed with paraformaldehyde and embedded by paraffin. Sections (5 μm) were used for PAS staining; glomerular size and mesangial expansion were assessed as previously described (22). Quantification was performed using ImageJ (NIH).

### Transmission electron microscopy.

The complete method is previously described (22, 61). Kidney tissue samples were fixed in 2.5% glutaraldehyde in 0.1M phosphate buffer and processed for electron microscopy. Images were obtained using Philips 301 Transmission Electron Microscope. For GBM measurement, there were 100 measurements from each mouse. In total, 27–30 electron micrographs of nonoverlapping fields were taken from each glomerulus at magnification of ×19,000. The number of filtration slits was obtained by counting the number of slit pores per 100 μm of GBM from each set of micrographs. Quantification was performed using ImageJ.

### PK activity assays.

We measured PK activity using the method described previously (22). Briefly, PK activity was measured from pyruvate-related conversion of NADH to NAD+ by lactate dehydrogenase (LDH). Cells or kidney tissues were lysis in PLB buffer (50 mM Tris-HCl [pH 7.5], 1 mM EDTA, 150 mM NaCl, 1% Igepal-630) with protease/phosphatase inhibitors. In total, 5 μg of cell lysate was mixed with PK reaction buffer (50 mM Tris-HCl [pH 7.5], 100 mM KCl, and 5mM MgCl_2_) containing 0.5 mM PEP (Sigma-Aldrich, P0564), 0.6mM ADP (Sigma-Aldrich, A5285), 180μM NADH (Sigma-Aldrich, N8129), and 160μg/mL LDH (Sigma-Aldrich, L1254) and read at excitation wavelength, 340 nm; emission wavelength, 460 nm.

### RNA isolation and quantitative PCR.

RNA was isolated using PureLink RNA Mini kit (Invitrogen). In total, 1 μg of RNA was used to generate cDNA using high-capacity cDNA reverse transcription kit (Applied Biosystems). Quantitative PCR (qPCR) was performed using PowerUp SYBR Green Master Mix (Applied Biosystems). Gene expression was normalized to 36B4 expression. Primer sequences are listed in Supplemental Table 2.

### Western blotting.

Cells or tissues were lysed in RIPA buffer (50 mM Tris-HCl, 150mM NaCl, 1% NP-40, 0.5% sodium deoxycholate, and 0.1% SDS). Protein concentration was determined by BCA protein assay (Thermo Fisher Scientific). Precast gels (4%–20% TGX precast gels; Bio-Rad) and PVDF membranes were used for electrophoresis and transfer. Membranes were blocked with 5% nonfat dry milk in Tris-buffered saline 0.1% Tween-20 for 1 hour and incubated with primary antibody in 4°C overnight. The membranes were then rinsed and incubated with corresponding HRP-conjugated secondary antibody. Detection was carried out using an ECL Detection kit (Thermo Fisher Scientific). Quantification was performed using ImageJ. Antibodies, along with manufacturer information, are listed in Supplemental Table 3.

### Glucose flux in mitochondrial and glycolysis.

Extracellular flux was measured using XF96 Extracellular Flux Analyzer. During Seahorse experiments, Agilent Seahorse XF DMEM Medium (pH 7.4) was used. OCR and extracellular acidification rate (ECAR) were measured using FluxPak mini kit (100867-100, Seahorse Biosciences). Glomeruli was harvested as described and was seeded in DMEM with 20% FBS, 5 mM glucose, no pyruvate in collagen I precoated 96-well plates for 24 hours before experimentation. The glomeruli were treated with mouse recombinant VEGF (493MV005, R&D Systems) 2 hours before assay, and VEGF neutralizing antibody (AF-493-NA, R&D Systems) was treated 24 hours before assay. For glycolysis stress test, no glucose, pyruvate, or glutamine was included in the assay medium at basal measurement, with additional injection of low glucose (5 mM), high glucose (20 mM), rotenone (1 μM), and 2-DG (50 mM) of working concentration. For mitochondrial stress test, 5 mM glucose was added to the assay medium at basal measurements followed by oligomycin (1 μM), FCCP (1.5 μM), and rotenone (1 μM).

### Plasmid site mutation.

QuikChange II XL Site-Directed Mutagenesis Kit (Agilent) was used for site mutation of human PKM2 plasmid according to manufacturer’s instruction. The sequence of mutagenic primers was forward, 3′-TTCTTGTAGTTCTAATAGTCGTACTAGCTCTTAGTACTCCCCCAAG-5′, reverse, 5′-AAGAACATCAAGATTATCAGCATGATCGAGAATCATGAGGGGGTTC-3′.

### Lentivirus packaging.

Lentiviral shRNAs mouse PKM (TRCN0000025621) and pLKO (scramble) were purchased from Thermo Fisher Scientific. Human PKM1 (catalog EX-Z5842-Lv152) and PKM2 (catalog EX-Z7438-Lv152) plasmid were purchased from Genecopoeia. phPKM1, phPKM2, and phPKM2(K270M) lentivirus were produced by general lentiviral transfection.

### Generation of podocyte stable cell lines.

pEmpty and pPKMKD stable cell lines were generated by pLKO and PKM shRNA lentivirus; cells were selected by puromycin for 10 days and verified by PKM1 and PKM2 protein levels and pk activity. PKM1 and PKM2 overexpression and reexpression stable cell lines were further generated by infecting phPKM1, phPKM2, and phPKM2(K270M) to pEmpty and pPKMKD podocyte stable cell lines. After infection, cells were selected by hygromycin for 10 days. All the podocyte stable cell lines were verified by measuring PKM1 and PKM2 protein level, as well as total pk activity.

### Cell culture.

Mouse podocyte cell lines were gifts from P. Mundel (Mount Sinai School of Medicine, New York, New York, USA). Podocytes were cultured in RPMI medium with 10% FBS, as previously described at 33°C with IFN-γ and then thermo-shifted to 37°C for 10 days without IFN-γ induction (13), and cells were incubated with DMEM with 1% FBS, 5 mM glucose, and an absence of pyruvate for 24 hours before experiments. Mouse primary cultured glomerular endothelial cells were purchased from Cell Biologics and used for experiments between passages 3 and 5.

### VEGF ELISA.

Isolated human glomeruli samples were lysis in RIPA buffer, the concentration of VEGF was measured from supernatant after centrifuge by human VEGF QuantiGlo ELISA Kit (R&D Systems). The concentration of mouse VEGF production in the cell culture medium was measured by mouse VEGF Quantikine ELISA Kit (R&D systems).

### Statistics.

Figures were produced using GraphPad prism software. All data are presented as either means ± SEM or ± SD in figure legends. Unpaired Student’s *t* tests with no assumption of equal variance were used for comparisons between 2 groups. To determine goodness of fit correlation (*R*^2^), univariate linear regression was used. For comparisons of more than 2 groups, 2-way ANOVA was used. Diabetic mice were excluded from the analysis if blood glucose was less than 400 mg/dL. For the Medalists’ clinical characteristics, statistical analyses were performed using SAS v. 9.4. Descriptive statistics are presented as mean (± SD), median (quartile 1 [Q1], quartile 3 [Q3]), or percentage (N%), as appropriate. Tests to determine between-group differences were applied using a 2-sample *t* test for continuous variables with a normal distribution; a Wilcoxon rank-sum test for continuous variables with a nonnormal distribution; and a χ^2^ test of independence or Fisher’s exact test for categorical variables. Statistical significance was set at *P <* 0.05.

### Study approval.

Human studies were approved by Joslin Diabetes Center committee on Human studies prior to commencement of the study. All mouse studies were approved by the IRB at Joslin Diabetes Center and some protocols were adapted from Animal Models of Diabetic Complications Consortium (AMDCC). Standards established by the Animal Welfare Acts, PHS Policy on Humane Care and Use of Laboratory Animals, and *Guide for the Care and Use of Laboratory Animals* (National Academies Press, 2011) were followed.

## Author contributions

JF, TS, QL, and GLK designed the experiments. JF and TS performed the experiments and interpreted the data. QL, RS, KP, MGY, HY, FS, QH, and IW assisted the experiments and data analysis. JF and GLK wrote the manuscript. TS, QL, RS, MGY, HY, FS, and QH helped interpret the results and assisted with manuscript preparation and editing. All authors edited and approved the final version. JF and TS contributed as co–first authors; authorship order is based on the amount of data contributed.

## Supplementary Material

Supplemental data

## Figures and Tables

**Table 1:**
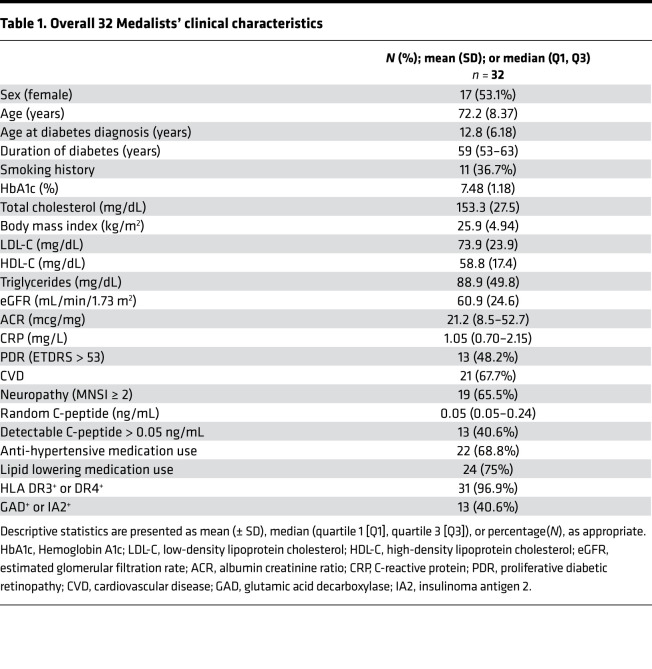
Overall 32 Medalists’ clinical characteristics

## Version 1 02/08/2022

In-Press Preview

## Version 2 03/08/2022

Electronic publication
