# Supplementary material for: Regeneration of glomerular metabolism and function by podocyte pyruvate kinase M2 in diabetic nephropathy
Source: JCI Insight. 2022 Mar 8;7(5):e155260. doi: 10.1172/jci.insight.155260 (PMC8983139; doi:10.1172/jci.insight.155260)
Supplement: Supplemental data [file jciinsight-7-155260-s182.pdf]

Supplemental Figure 1

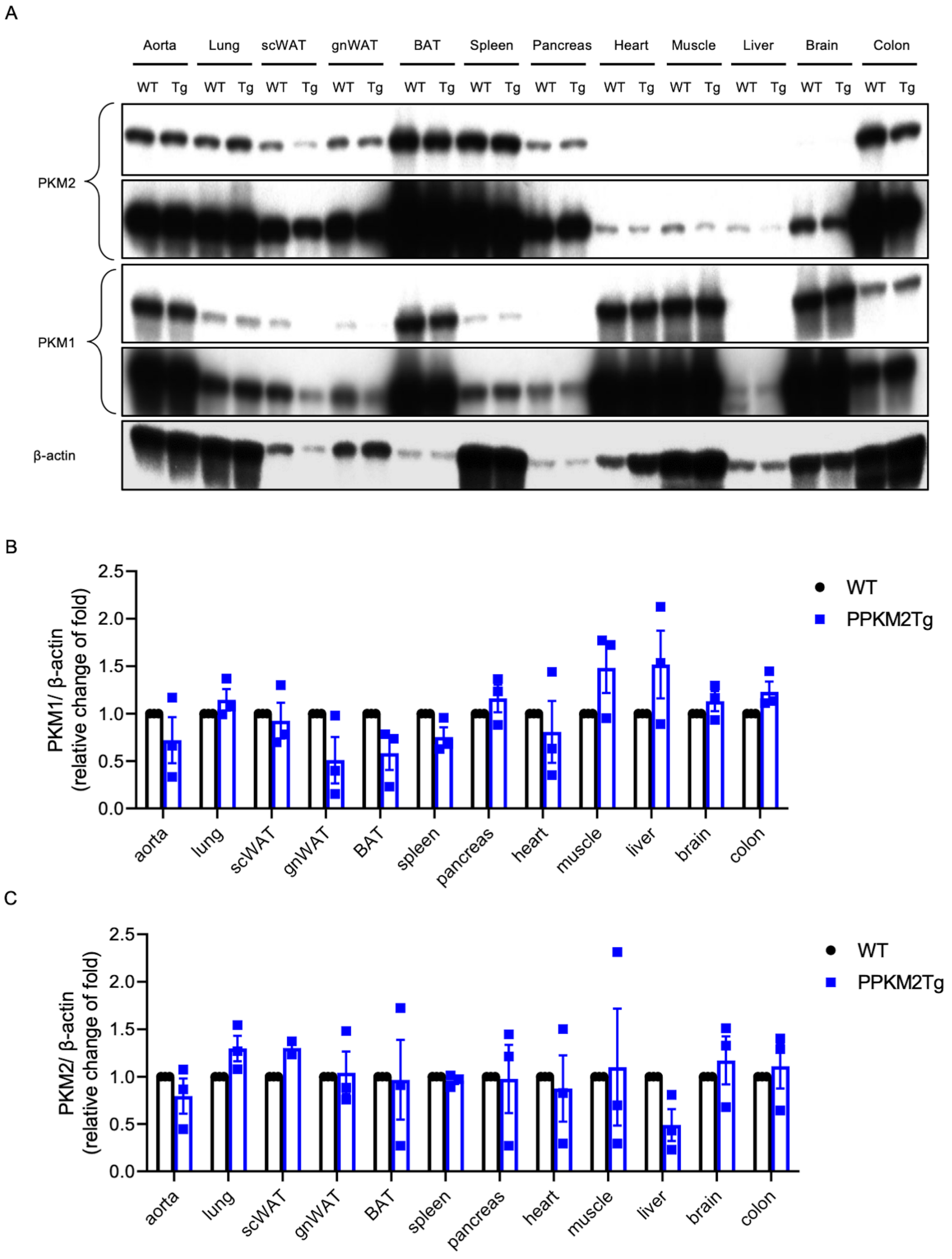

Supplemental Figure 1. PKM2 expression from different tissues of PPKM2Tg mice.
(A, B) PKM2 and PKM1 expression levels in different tissues of WT and PPKM2Tg mice were determined by Western blotting (n = 3 per group). Data are mean ± SEM.

Supplemental Figure 2

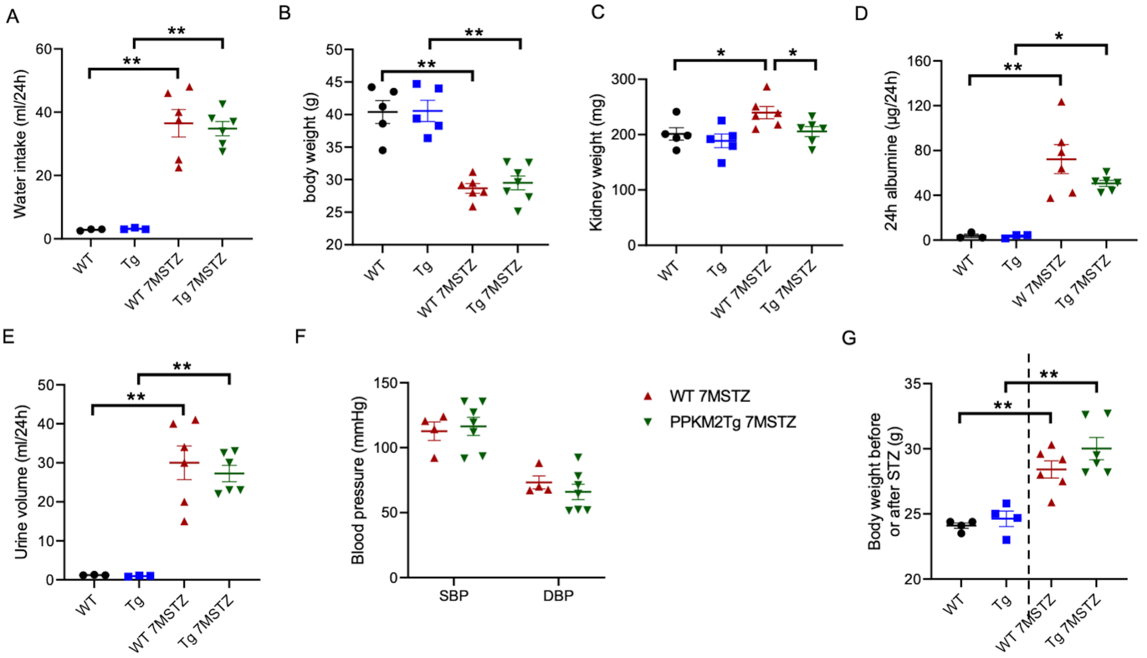

Supplemental Figure 2. Metabolic parameters of PPKM2Tg 7 months post-STZ.
(A) Water intake of PPKM2Tg and WT mice 7 months post-STZ. **(B)** Body weight of PPKM2Tg and WT mice 7 months post-STZ. **(C)** Kidney weight of PPKM2Tg and WT mice 7 months post-STZ. **(D)** 24-hour albumin of PPKM2Tg and WT mice 7 months post-STZ. **(E)** 24-hour urine volume of PPKM2Tg and WT mice 7 months post-STZ. **(F)** Blood pressure of PPKM2Tg and WT mice 7 months post-STZ. **(G)** Body weight of PPKM2Tg and WT at 8 weeks and 7 months post-STZ. Nondiabetic WT mice (n = 3); PPKM2Tg mice (n = 3); WT 7MSTZ mice (n = 6); PPKM2Tg 7MSTZ mice (n = 6). * $p < 0.05$; ** $p < 0.01$; *** $p < 0.001$. Data are mean ± SEM, two-way ANOVA followed by correction for multiple comparison.

Supplemental Figure 3

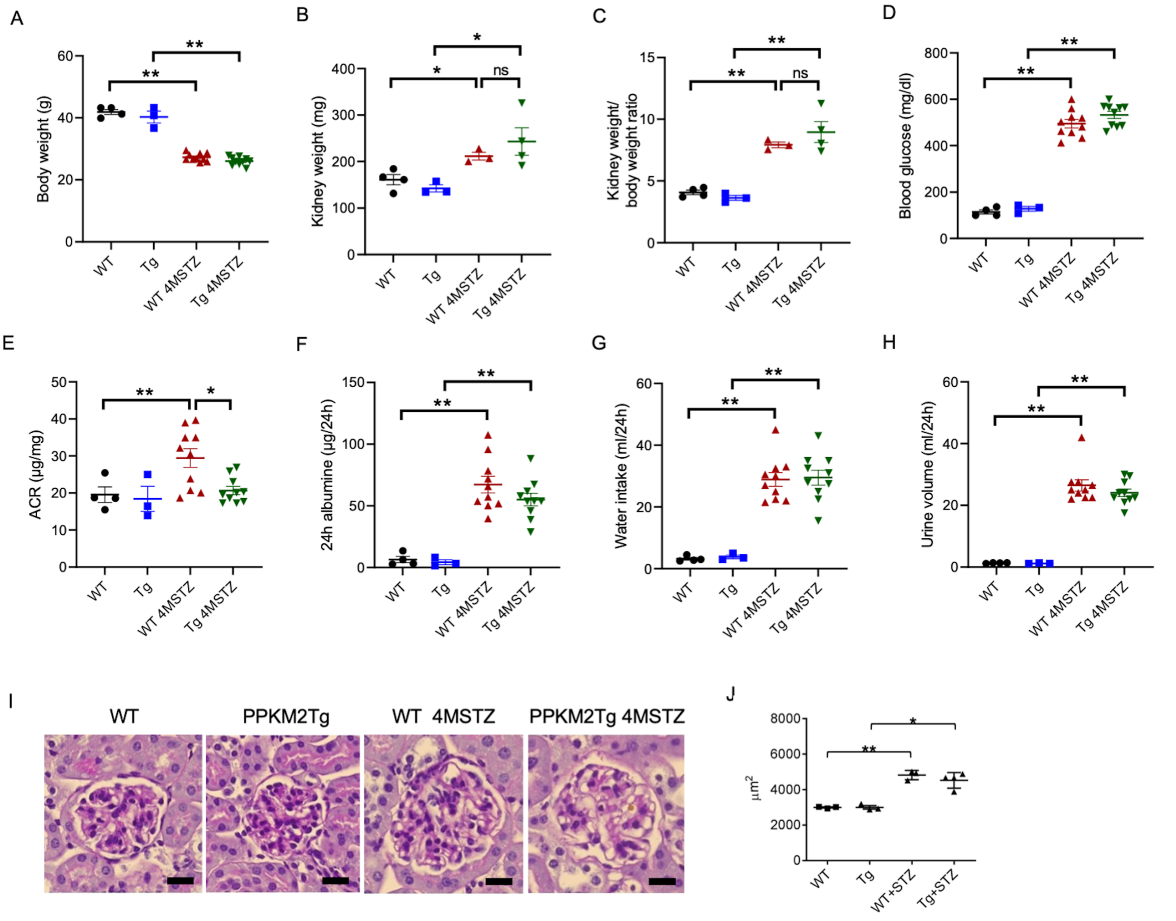

Supplemental Figure 3. Metabolic parameters of PPKM2Tg 4 months post-STZ.
(A) Body weight of PPKM2Tg (Tg 4MSTZ) and WT 4 months post-STZ (WT 4MSTZ). **(B)** Kidney weight of PPKM2Tg and WT 4 months post-STZ. **(C)** Kidney weight/ body weight ratio 4 months post-STZ. **(D)** Fasting blood glucose 4 months post-STZ. **(E)** Albumin creatinine ratio 4 months post-STZ. **(F)** 24-hour albumin of PPKM2Tg and WT 4 months post-STZ. **(G)** Water intake of PPKM2Tg and WT 4 months post-STZ. **(H)** 24-hour urine volume of PPKM2Tg and WT 7 months post-STZ. Nondiabetic WT mice (n = 4); PPKM2Tg mice (n = 3); WT 4MSTZ mice (n = 10); PPKM2Tg 4MSTZ mice (n = 10). * $p < 0.05$; ** $p < 0.01$; *** $p < 0.001$. **(I)** Representative image of glomeruli 4 months post-STZ by PAS staining. n = 10-20 images of PAS-stained kidney sections for each mouse. Scale bars, 20µm. **(J)** Glomerular size 4 months post-STZ. Nondiabetic WT mice (n = 3); PPKM2Tg mice (n = 3); WT 4MSTZ mice (n = 4); PPKM2Tg 4MSTZ mice (n = 4). * $p < 0.05$; ** $p < 0.01$. Data are mean ± SEM, two-way ANOVA followed by correction for multiple comparison.

Supplemental Figure 4

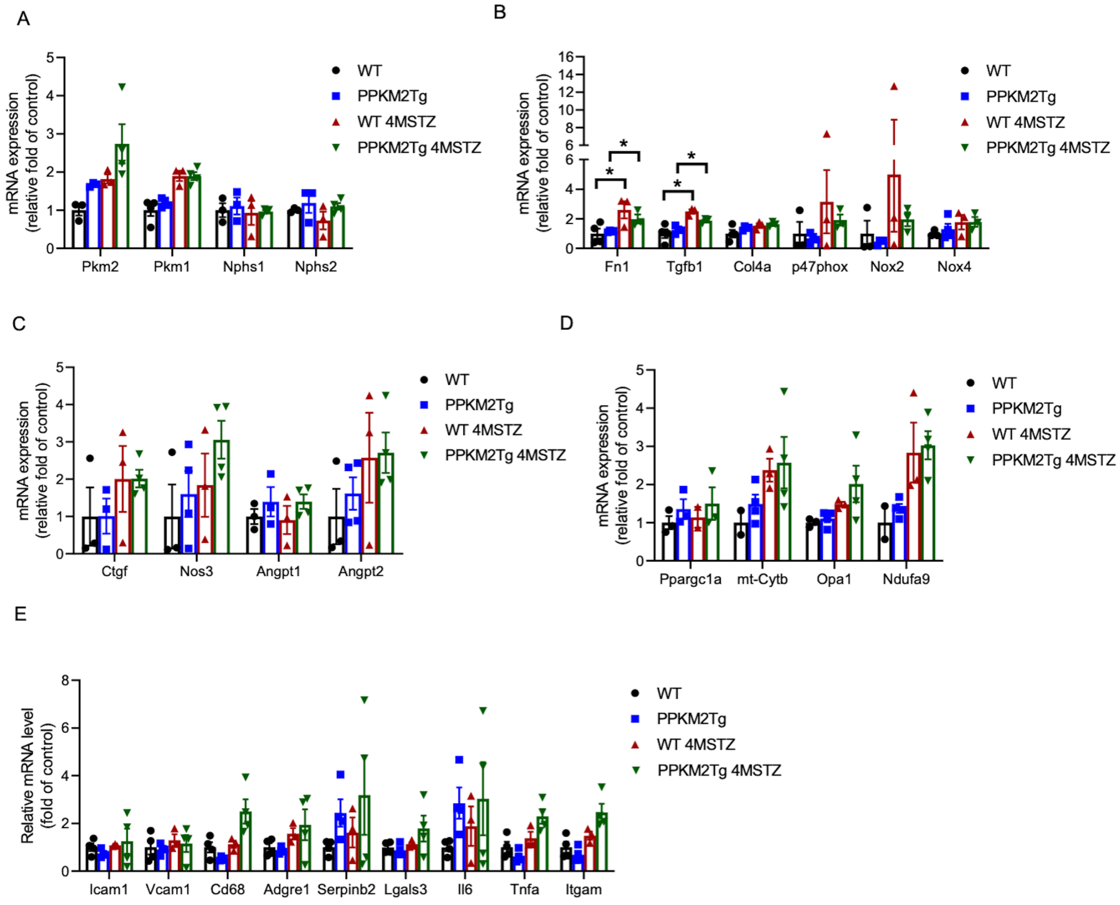

Supplemental Figure 4. Gene expression in the glomeruli of PPKM2Tg 4 months post-STZ. (A) mRNA level of PKM1, PKM2 and podocyte markers in the glomeruli of diabetic PPKM2Tg mice 4 months post-STZ. **(B)** Fibrotic and oxidative stress gene expressions in the glomeruli of PPKM2Tg 4 months post-STZ. **(C)** mRNA expression of endothelial trophic genes in the glomeruli of diabetic PPKM2Tg mice. **(D)** mRNA of mitochondrial related genes in the glomeruli of PPKM2Tg 4 months post-STZ. **(E)** mRNA expression of inflammatory genes in the cortex of diabetic PPKM2Tg mice. WT mice (n = 4); PPKM2Tg mice (n = 4); WT 4MSTZ mice (n = 3); PPKM2Tg 4MSTZ mice (n = 4). * $p < 0.05$. Data are mean ± SEM, two-way ANOVA followed by correction for multiple comparison.

Supplemental Table 1. Medalists' clinical characteristics in DN non-protected (n = 10) and DN protected (n = 11) groups

	**DN non-protective**<br>eGFR<45 ml/min/1.73 m2 (n=10)<br>%; median (Q1, Q3)	**DN protective**<br>eGFR>70 ml/min/1.73 m2 (n=11)<br>%; median (Q1, Q3)	**p**
Sex (female)	8 (80%)	4 (36.4%)	0.08
Age (years)	71.8 (6.09)	68.1 (8.54)	0.27
Age at diabetes diagnosis (years)	15.6 (6.42)	10.9 (6.24)	0.11
Duration of diabetes (years)	57.5 [53-59]	55 [51-61]	0.75
HbA1c (%)	7.69 (1.19)	7.32 (1.37)	0.52
Total cholesterol (mg/dL)	140.5 (20.2)	161.9 (36.2)	0.12
Body mass index (kg/m2)	25.4 (3.85)	27.2 (7.04)	0.53
LDL-C (mg/dL)	66.2 (21.6)	76.7 (27.8)	0.36
HDL-C (mg/dL)	51.7 (13.1)	63.7 (18.2)	0.10
Triglycerides (mg/dL)	95.5 (40.5)	75.8 (56.7)	0.38
eGFR (mL/min/1.73m2)	30.7 (10.6)	86.5 (8.30)	<0.0001
Glomerular VEGF (pg/mg)	288.6[21.61-608.2]	672.7[299.4-1128]	0.0026
ACR (mcg/mg)	17.5 [7.25-166]	15.8 [11.8-37]	1
CRP (mg/L)	1.35 [0.84-2.2]	1.1 [0.63-2.1]	0.57
PDR (ETDRS>53)	3 (37.5%)	5 (55.6%)	0.64
CVD	7 (70%)	6 (54.6%)	0.66
Neuropathy (MNSI ≥2)	5 (50%)	6 (66.7%)	0.65
Random C-peptide (ng/mL)	0.17 [0.05-0.36]	0.05 [0.05-0.24]	0.35
Detectable C-peptide>0.05 ng/mL	7 (70%)	4 (36.4%)	0.2
Anti-hypertensive medication use	7 (70%)	6 (54.6%)	0.66
HLA DR3 or DR4-positive	10 (100%)	10 (90.9%)	1
GAD or IA2-positive	4 (40%)	4 (36.7%)	1

Descriptive statistics are presented as mean (+ SD), median (quartile 1 [Q1], quartile 3 [Q3]), or percentage (N), as appropriate.

Supplemental Figure 5

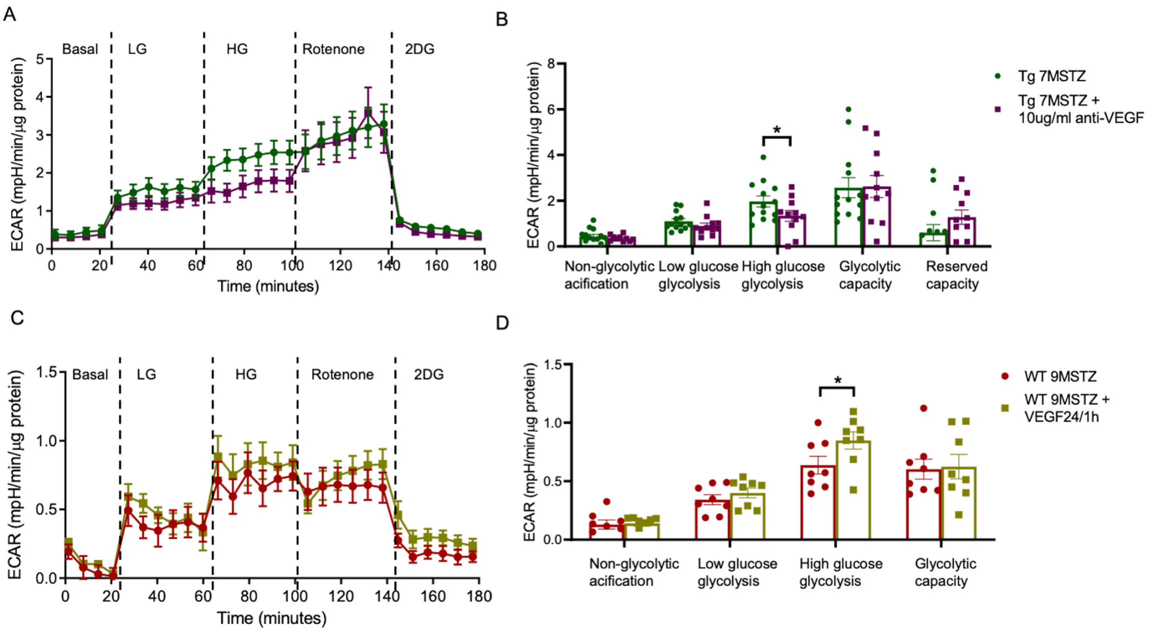

Supplemental Figure 5. VEGF regulation of glycolytic rate in diabetic mice 7 months post-STZ.

Glomeruli from diabetic PPKM2Tg mice were treated with anti-VEGF (10µg/ml) for 24 hours; representative curve of ECAR and quantitated data were shown in **(A)** and **(B)**. Tg 7MSTZ (n = 7); Tg 7MSTZ + anti-VEGF (n = 10). * $p < 0.05$. Diabetic WT mice 7-9 months post STZ (7-9MSTZ) were incubated with mVEGF (100ng/ml) for 24 hours and addition of mVEGF (100ng/ml) 1 hour before Seahorse assay; representative curve of ECAR and quantitated data were shown in **(C)** and **(D)**. WT 7-9MSTZ (n = 7); WT 7-9MSTZ + VEGF 24/1h (n = 8). * $p < 0.05$. Data are mean ± SEM.

Supplemental Figure 6

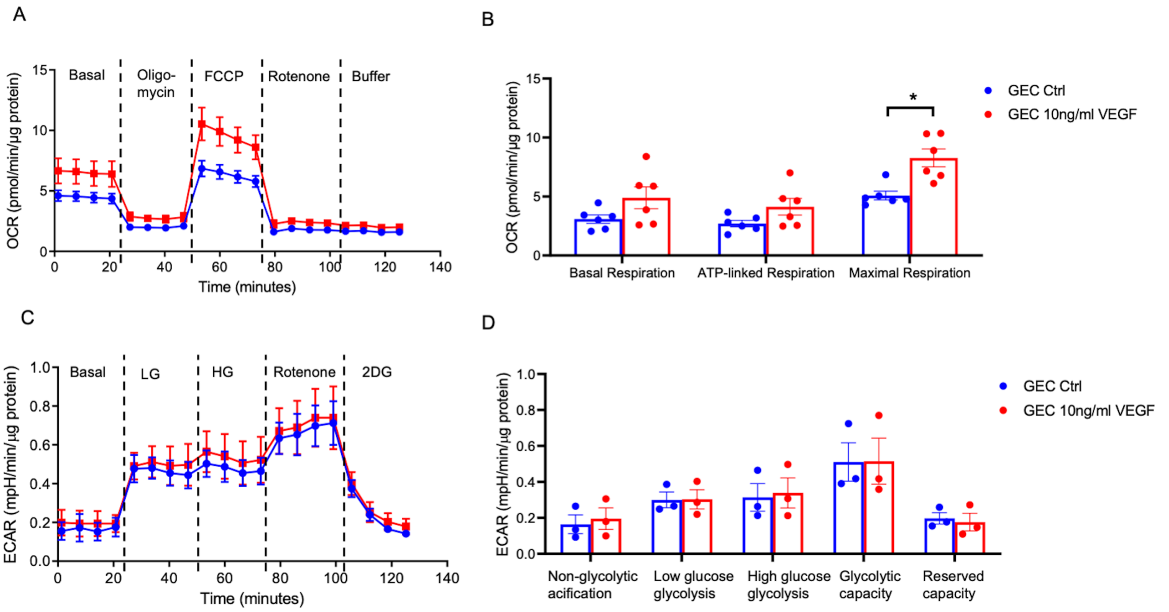

Supplemental Figure 6. VEGF regulation of glycolytic rate in primary cultured glomerular endothelial cells.

Primary cultured glomerular endothelial cells were incubated with mVEGF (10ng/ml) for 1 hour before Seahorse assay; OCR**(A)** and ECAR **(C)** were measured and quantitated data were showed in **(B)** and **(D)**. For OCR **(A, B)**, n = 6; * $p < 0.05$. For ECAR **(C, D)**, n = 3. Data are mean ± SEM.

Supplemental Table 2. List of primer sequences for RT-PCR

Gene	Forward (5' – 3')	Reverse (5' – 3')
Pkm1	GCTGTTTGAAGAGCTTGTGC	TTATAAGAGGCCTCCACGCT
Pkm2	TGTCTGGAGAAACAGCCAAG	TCCTCGAATAGCTGCAAGTG
Nphs1	ACCTGTATGACGAGGTGGAGAG	ACCTGTATGACGAGGTGGAGAG
Nphs2	GTCCTCGCCTCCCTGATCTT	GTCCTCGCCTCCCTGATCTT
Fn1	CGAGGTGACAGAGACCACAA	CTGGAGTCAAGCCAGACACA
Tgfb1	CCTGAGTGGCTGTCTTTTGA	CGTGGAGTTTGTTATCTTTGCTG
Col4a	GACAGCCAGGTTTGACAGGT	GGCAGCTCTCTCCTTTCTGA
Ctgf	GTCTTCACACTGGTGCAGCC	ACTGGAAGACACATTTGGCC
p47phox	TCCCAACTACGCAGGTGAAC	CCTGGGTTATCTCCTCCCCA
Nox2	CCTGGGTTATCTCCTCCCCA	AGATGTGCAATTGTGTGGATGGCG
Nox4	TGTTGGGCCTAGGATTGTGTT	AAAAGGATGAGGCTGCAGTTG
Hif1a	GGATGAGTTCTGAACGTCGAAA	ACATTGTGGGGAAGTGGCAA
Vegf	CTCGCAGTCCGAGCCGGAGA	GGTGCAGCCTGGGACCACTTG
Nos3	GAAGGCTTTTGATCCCCGGGTCCTG	CAGTTCCTCCAGCCGTGTGTCCAC
Angpt1	AAATTATACTCAGTGGCTGGAA	TTCTAGGATTTTATGCTCTAATAA
Angpt2	AGAGTATTGGCTGGGCAACGAGTT	TCCTTTGTGCTAAAATCACTTCCT
Ppargc1a	GTCAACAGCAAAAGCCACAA	TCTGGGGTCAGAGGAAGAGA
Opa1	GTGTGCTGGAAATGATTGCTC	TGGTGAGATCAAATTCCCGAG
mt-Cytb	TATCGCGGTCCTAGCAATCG	ATGGGGTCGGGTGTTTAGTG
Ndufa9	CCACCTCGAGTCATGGTGTA	TTCAAACCGGGCTCTCATCA
Ccl2	GTCCCTGTCATGCTTCTGG	GCTCTCCAGCCTACTCATTG
Tnfa	GCCACCACGCTCTTCTGTCT	GTCTGGGCCATAGAACTGAT
Il1b	TCCCGTGGACCTTCCAGGATGAG	TCGGAGCCTGTAGTGCAGTTGTC
Adgre1	ACCACAATACCTACATGCACC	AAGCAGGCGAGGAAAAGATAG
Itgam	ATGGACGCTGATGGCAATACC	TCCCCATTCACGTCTCCCA
36b4	GCTCCAAGCAGATGCAGCA	CCGGATGTGAGGCAGCAG

Supplemental Table 3. List of antibodies

Antibodies	Catalogue number	Source	Concentration
PKM1	7067S	Cell Signaling Technology	1:1000 dilution
PKM2	4053S	Cell Signaling Technology	1:1000 dilution
Nephrin	ab183099	Abcam	1:1000 dilution
Podocin	sc-21009	Santa Cruz Biotechnology	1:1000 dilution
AQP1	sc-25287	Santa Cruz Biotechnology	1:1000 dilution
Actin	sc-47778 HRP	Santa Cruz Biotechnology	1:100,000 dilution
p-eNOS(Ser1177)	BDB612392	BD Biosciences	1:1000 dilution
eNOS	BDB610297	BD Biosciences	1:1000 dilution
Anti-mouse IgG	sc-516102	Santa Cruz Biotechnology	1:5000 dilution
Anti-rabbit IgG	sc-2357	Santa Cruz Biotechnology	1:5000 dilution
